# A Three-Module Machine Learning Framework for Protein Sequence- and Temperature-Dependent *k*_cat_/*K*_m_ Prediction in β‑Glucosidases

**DOI:** 10.1021/acssynbio.5c00257

**Published:** 2025-10-02

**Authors:** Mehmet Emre Erkanli, Yunseok Jang, Ali Malli, Khalid El-Halabi, Chaehyun Ryu, Jin Ryoun Kim

**Affiliations:** 1 Department of Chemical and Biomolecular Engineering, 5894 New York University, 6 MetroTech Center, Brooklyn, New York 11201, United States; 2 Center for Data Science, New York University, 60 5th Ave, New York, New York 10011, United States

**Keywords:** enzyme, *k*_cat_/*K*_m_, machine learning, β-glucosidase

## Abstract

The catalytic activity of enzymes is intricately determined by their amino acid sequences and assay conditions, particularly temperature. Navigating the complex interplay among sequence, temperature, and catalytic function is crucial for unlocking a multitude of enzyme applications. Machine learning has recently emerged as a tool for quantitative prediction of enzyme activity from protein sequences. Unfortunately, ML models designed to predict the comprehensive enzyme activity parameter, *k*_cat_/*K*_m_, from protein sequences are rare compared to those predicting *k*_cat_ or *K*_m_ alone. Combining both protein sequence and temperature as input features further challenges predictions; no current ML models capture the nonlinear relationship between *k*_cat_/*K*_m_ and temperature for a protein sequence of interest. In this study, we developed a unique three-module ML framework that predicts β-glucosidase *k*_cat_/*K*_m_ values based on protein sequence and temperature. Each module was designed to capture a distinct aspect of the interplay among protein sequence, temperature, and *k*_cat_/*K*_m_ for β-glucosidase activity; when integrated, they formed an ML framework that maps the sequence and temperature spaces associated with β-glucosidase *k*_cat_/*K*_m_. This modular approach allowed for optimizations of ML models within each module, collectively achieving notable generalization performance when predicting temperature-dependent *k*_cat_/*K*_m_ values for protein sequences not encountered during training. Our findings underscore the advantages of the three-module framework over traditional single-module methods, particularly by reducing prediction variability due to data splitting and mitigating overfitting. We anticipate that our multimodule ML framework will be directly applicable to other complex systems, enabling quantitative exploration of their property domains.

## Graphical abstract

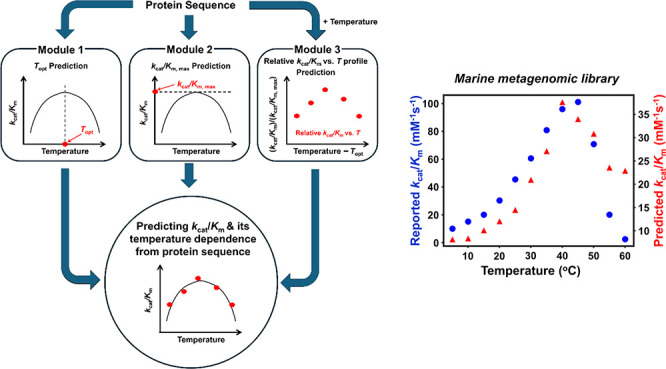

## Introduction

Establishing sequence–function relationships is key to annotating and evolving enzymes for specific applications.^1^ In particular, catalytic functions are primarily considered in the search for enzymes suitable for specific applications. Unfortunately, interrogating the functional outcomes of enzymes across sequence variations is highly challenging due to the high dimensionality of sequence space.^2^ For example, there are 20^320^ possible sequence variations for enzymes with 320 amino acids, which is a typical size for bacterial proteins.^3^ Even enzymes with the same catalytic functions originate from a diverse range of species and vary in amino acid sequence, composition, and length. Besides the sequence space, functional landscapes of enzymes are also shaped by environmental factors (e.g., temperature).^4^ Thus, an intricate interplay between intrinsic and extrinsic factors is involved, as demonstrated with thermal adaptation of thermophilic, mesophilic, and psychrophilic enzymes.^5^

Experimental exploration of an enzyme’s sequence space typically involves point mutations.^6^ Unfortunately, navigating nonlocal sequence space experimentally is highly resource-intensive.^7,8^ Typical mutagenesis-based experiments utilize only a few mutational steps within a small region of latent sequence space.^9^ Functional evaluations within such narrow sequence spaces often fail to extrapolate when establishing sequence–function relationships due to the nonadditivity of functional mutations and/or epistatic mutational effects.^10,11^ Instead, computational searches can efficiently map nonlocal sequence space, extending beyond the reach of experimental methods confined to local sequence space.^12,13^ However, despite advances in computing protein stability, solubility, and expression levels, developing a computational tool for predicting the catalytic functions of enzymes remains challenging. This is due to the limited understanding of how an enzyme’s sequence impacts its catalytic function. Catalytic function involves complex multistep mechanisms and molecular interactions,^14^ which are encoded globally across the protein chain.^15,16^ The catalytic function is also influenced by extrinsic factors, adding complexity to the prediction of sequence–function–environment relationships.

Over the past decades, machine learning (ML) has emerged as a computational tool for predicting structure, stability, solubility, and function of proteins.^7,17–22^ Machine learning extracts rich information from raw inputs and provides a good estimate of the functional characteristics. Many ML models were constructed to predict protein functions from protein structures.^23,24^ However, these structure-based ML approaches face challenges in accurately computing function-relevant conformational dynamics, which are crucial for the catalytic functions of enzymes,^25,26^ from static structures. Even similarly structured enzymes often display very different enzyme activity.^27^ Instead, ML models trained on protein sequences and their functions can infer functional characteristics of unseen sequences without prior knowledge of the protein structures or the underlying molecular mechanisms.^7,28^ Sequence-based ML models have been shown to determine epistatic interactions,^29^ classify protein families,^30^ assess protein stability,^31,32^ and rationalize mutational effects on enzyme activity,^18^ enzyme enantioselectivity,^33^ and ligand binding affinity^32^ from protein sequences as inputs.

To fully utilize the wealth of information with minimal lab-to-lab variations, an ML model would significantly benefit from data on standardized parameters related to key catalytic activities (e.g., *k*_cat_, *K*_m_, and *k*_cat_/*K*_m_). Compared to *k*_cat_ and *K*_m_, *k*_cat_/*K*_m_ offers the most comprehensive enzymatic information and serves as a standardized measure of catalytic proficiency.^34,35^ Unfortunately, ML models for *k*_cat_/*K*_m_ prediction from protein sequence are rare, compared to those for *k*_cat_ or *K*_m_,^36–41^ likely due to the smaller dataset and the more complex nature of *k*_cat_/*K*_m_.^38,42^ Additionally, using separate ML models to estimate *k*_cat_ and *K*_m_ individually for calculating the *k*_cat_/*K*_m_ ratio introduces inaccuracies due to error propagation.^38^ A previous *k*_cat_/*K*_m_ ML model (named as the UniKP model) was built on a dataset comprising 910 entries curated from the public database, which covers various combinations of enzyme sequences, substrate structures, and their corresponding *k*_cat_/*K*_m_ values.^38^ This *k*_cat_/*K*_m_ ML model achieved a coefficient of determination (*R* ^2^) of 0.65 when the validation sets contained a number of protein sequences that overlapped with those in the training set.^38^ Later, another ML model predicting *k*_cat_/*K*_m_ through transfer learning from *k*_cat_ and *K*_m_ models (named as the EITLEM-Kinetics model) was developed using a larger dataset (*N* = 13 388).^42^ This model achieved an *R* ^2^ of 0.519 when evaluated on validation sets with no overlapping entries from the training sets used for the *k*_cat_ and *K*_m_ models. The *R* ^2^ of this model increased to 0.680 after eight iterations of transfer learning among the *k*_cat_, *K*_m_, and *k*_cat_/*K*_m_ models.^42^ CataPro achieved a Pearson correlation coefficient (PCC) of 0.41 in predicting *k*_cat_/*K*_m_, through transfer learning from its *k*_cat_ and *K*_m_ models.^43^

Since environmental conditions (extrinsic factors) often impact catalytic activity more than sequence variations (intrinsic factors),^44^ training ML models with both is crucial for accurate functional annotation.^38^ Regarding the extrinsic factors, temperature is one of the most significant parameters, as it can change *k*_cat_/*K*_m_ by several orders of magnitude. Additionally, enzyme processes are often carried out at nonoptimum temperatures due to the various advantages (e.g., increased substrate solubility at high temperatures and reduced enzyme aggregation at low temperatures).^45,46^ Unfortunately, most ML models for *k*_cat_, *K*_m_, or *k*_cat_/*K*_m_ prediction were built on data measured at a single temperature, limiting their applications. The EF-UniKP model, a derivative of the UniKP model, incorporates temperature as an additional feature for *k*_cat_ prediction.^38^ However, accurately predicting both *k*_cat_ and its temperature dependency has proven to be highly challenging:^38^ the *R* ^2^ for the temperature-dependent *k*_cat_ prediction was 0.38 on the validation set, which further decreased to 0.31 on a subset of validation set that contained either protein sequence or substrate not present in the training set.^38^ Additionally, it remains unclear whether this ML model can predict a complete, nonlinear *k*_cat_–temperature profile.

In the study presented here, we report on the construction of a three-module ML framework that collectively predicts *k*_cat_/*K*_m_ values as target regression variables from protein sequences and temperature as input variables. To better capture sequence and temperature spaces for specific functions, our model leverages smaller, specifically curated datasets from multiple sources, including literature, public databases, and our own experiments. This contrasts with broad-coverage models trained on large, functionally unrelated protein sequences from public databases, where the scarcity of *k*_cat_/*K*_m_–temperature data makes accurate modeling challenging. Our ML framework was specifically developed for β-glucosidase (BGL), one of the largest glycoside hydrolase families, as a model enzyme family. Our ML framework consisted of three independent ML modules for predicting (1) the optimum temperature (*T*_opt_), (2) *k*_cat_/*K*_m_ at *T*_opt_ (*k*_cat_/*K*_m,max_), and (3) the normalized *k*_cat_/*K*_m_ at temperatures relative to *T*_opt_ (referred to as a relative *k*_cat_/*K*_m_ vs *T* profile). The ML models in each module showed high generalization performance with BGL sequences that were unseen during the training (*R* ^2^ ∼ 0.6–0.85). These three modules were then integrated under the framework for the task of predicting *k*_cat_/*K*_m_ values as a function of the temperature for a protein sequence of interest. Our ML framework exhibited notable predictive performance with BGL sequences not seen during training, achieving an *R* ^2^ value of ∼0.38 for *k*_cat_/*K*_m_ values across different temperatures and protein sequences. We also demonstrate that a complete, nonlinear *k*_cat_/*K*_m_ vs temperature profile can be predicted using our ML framework for a given protein sequence. A traditional single-module ML model was constructed as a control using identical protein sequence representations, regression algorithms, and datasets. In comparison, the three-module ML framework provided more consistent prediction accuracy across different datasets while mitigating overfitting issues. Our results highlight that this unique three-module ML framework may serve as a valuable architecture for predicting highly complex catalytic variables across protein sequence and temperature spaces.

## Results

### Overview of the Three-Module ML Framework

Our three-module ML framework was designed to predict enzyme activity from the amino acid sequence and temperature as inputs. β-Glucosidase was chosen as a target enzyme activity for the ML framework, as this group of enzymes consists of proteins over a diverse sequence space, typically differing by up to hundreds of substitutions, deletions, and/or additions. Moreover, β-glucosidase plays key roles in various biotechnological sectors, including the biofuel, food, beverage, and pharmaceutical industries.^47^ Therefore, an ML-based activity prediction model for this enzyme would be highly valuable. An amino acid sequence was taken as a primary intrinsic parameter to determine the enzymatic activity (*k*_cat_/*K*_m_). Temperature can significantly influence enzymatic activity, often more so than sequence variations. Therefore, both the protein sequence and temperature were considered as input variables, with *k*_cat_/*K*_m_ as the target regression variable. We did not include protein structure as an additional feature, as it does not necessarily improve prediction accuracy.^41^ The effect of pH was not considered, primarily because modulating enzyme activity *in vitro* is more commonly achieved through temperature adjustments than through pH changes.

Our ML framework consisted of three independent modules for machine learning: the first for predicting the optimum temperature (referred to as *T*_opt_), the second for predicting *k*_cat_/*K*_m_ at *T*_opt_ (referred to as *k*_cat_/*K*_m,max_), and the third for predicting the temperature dependence of *k*_cat_/*K*_m_ relative to the *k*_cat_/*K*_m,max_ at *T*_opt_ (referred to as a relative *k*_cat_/*K*_m_ vs *T* profile) (Figure 1). For Modules 1 and 2 (i.e., the *T*_opt_ and *k*_cat_/*K*_m,max_ modules), the input feature was protein sequences. For Module 3 (i.e., the relative *k*_cat_/*K*_m_ vs *T* profile module), the input features were protein sequences and temperatures, and the output features were normalized *k*_cat_/*K*_m_ values at relative temperatures. Thus, each of the three modules was designed to learn about specific aspects of catalytic properties, collectively providing information about sequence- and temperature-dependent *k*_cat_/*K*_m_ values. For Module 2, logarithmic transformation was applied to the *k*_cat_/*K*_m,max_ values. As a control, we built a single-module framework using the same dataset.

**Figure 1:**
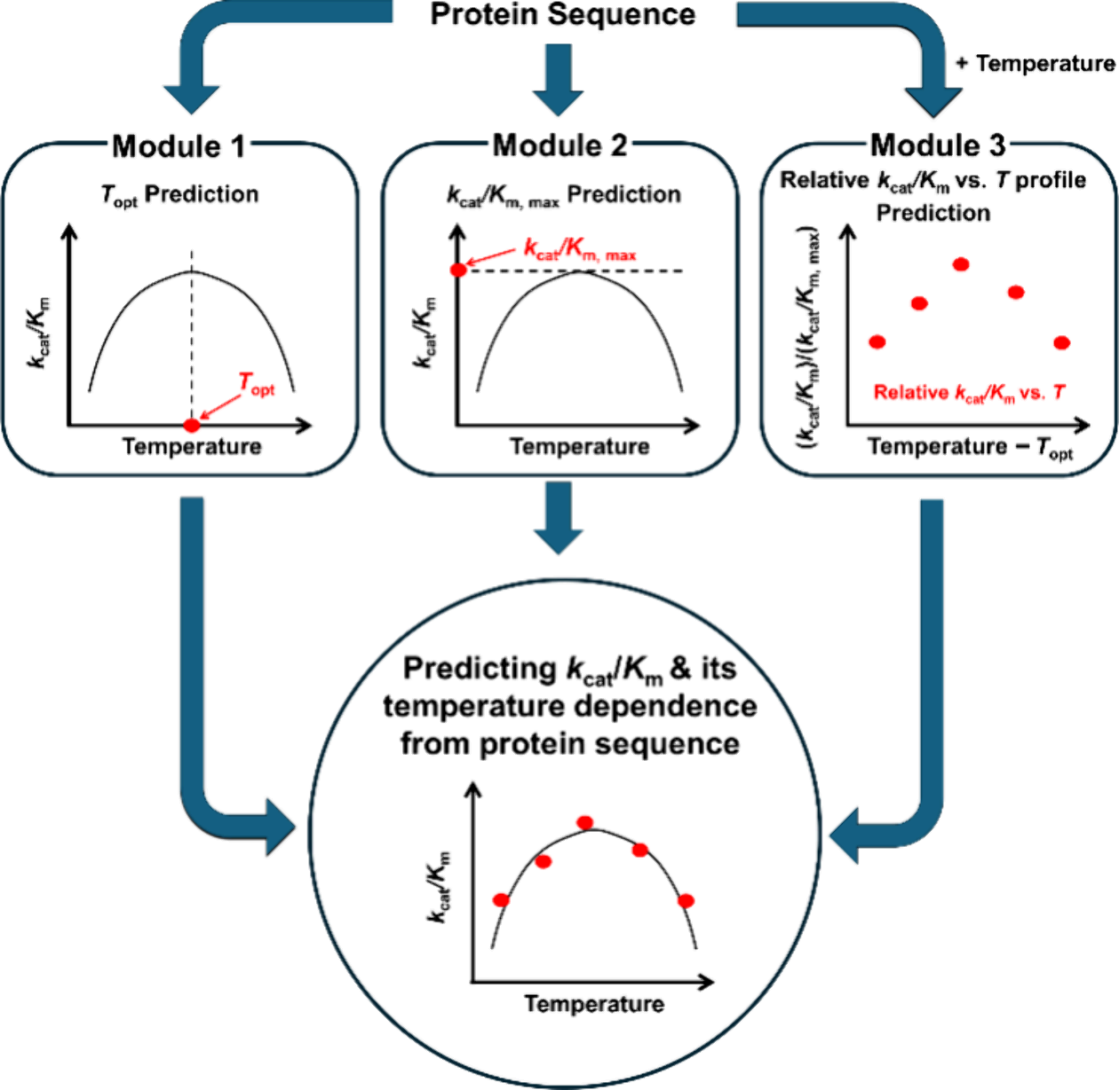
Overview of the three-model ML framework for prediction of *k*_cat_/*K*_m_ from amino acid sequence and temperature. The ML models in each of the three modules were trained to learn specific aspects of catalytic properties. The first model predicts *T*_opt_, the second predicts *k*_cat_/*K*_m,max_, and the third predicts a relative *k*_cat_/*K*_m_ vs *T* profile. When combined, the ML models in these three modules predict sequence- and temperature-dependent *k*_cat_/*K*_m_ values.

For the ML models in each module, we employed several protein sequence representation methods to convert amino acid sequences of enzymes into numerical vectors as input features. These methods ranged from one-hot-encoding, BLOcks SUbstitution Matrix (e.g., BLOSUM45), and overlapping *n*-gram representations (where *n* = 1, 2, and 3) to ESM1b, one of the largest protein language models.^48,49^ For Module 3, the components of each numerical vector representing an enzyme sequence were concatenated with the temperatures at which the *k*_cat_/*K*_m_ values were determined. The resulting concatenated vector was subsequently used as an input feature. For these ML models, the various regression algorithms such as Least Absolute Shrinkage and Selection Operator (LASSO), Random Forest (RF), and Extreme Gradient Boosting (XGB) were used.

### Dataset Construction

Our ML framework was created for β-glucosidase (BGL), an enzyme that hydrolyzes the β-1,4 glycosidic bond of cellobiose and small cellooligosaccharides. The scarcity of *k*_cat_/*K*_m_–temperature data in public databases containing functionally unrelated protein sequences makes accurate modeling challenging. To better capture sequence and temperature spaces for BGL activity, our model leverages smaller datasets curated from multiple sources (including literature, public databases, and our own experiments) targeted at BGLs from various organisms and biological kingdoms (Supporting Information, Tables S1 and S2 and Figure S1). Among the data reported with various BGL substrates, *k*_cat_/*K*_m_ values measured with *p*-nitrophenyl β-d-glucopyranoside (pNP-Glc) were collected due to the abundance of available information.

From the entire dataset, a subset was created by listing each protein sequence along with the temperature at which its highest *k*_cat_/*K*_m_ value was reported (Figure S2). This subset, referred to as the *T*_opt_ dataset (*N* = 260), was used to construct and validate ML models for predicting *T*_opt_ values from protein sequences. Another subset was compiled by listing each amino acid sequence and its highest *k*_cat_/*K*_m_ value at *T*_opt_ (Figure S2). This subset was used to build and validate ML models for predicting *k*_cat_/*K*_m,max_ values from amino acid sequences and was referred to as the *k*_cat_/*K*_m,max_ dataset (*N* = 260). For a BGL sequence with *k*_cat_/*K*_m_ measured at a single temperature, the measured temperature was taken as *T*_opt_ and the corresponding *k*_cat_/*K*_m_ value was considered as *k*_cat_/*K*_m,max_ for that sequence. Finally, the entire dataset was converted into one containing *k*_cat_/*K*_m_ values normalized by *k*_cat_/*K*_m,max_ for each protein sequence at temperatures relative to its *T*_opt_ (referred to as the relative *k*_cat_/*K*_m_ vs *T* profile dataset; *N* = 885; Figure S2). This converted dataset was used to develop and validate ML models for predicting relative *k*_cat_/*K*_m_ vs *T* profiles from protein sequences and temperatures.

### Evaluation of the Predictive Power of ML Models in Each Module on Unseen Sequences

To evaluate the robustness of models in each module of the framework, we conducted 5-fold cross-validation on the entire *T*_opt_, *k*_cat_/*K*_m,max_, and relative *k*_cat_/*K*_m_ vs *T* profile datasets, for all tested combinations of sequence representations and regression algorithms (Figure 2). It should be noted that the data entries in the *T*_opt_, *k*_cat_/*K*_m,max_, and relative *k*_cat_/*K*_m_ vs *T* profile datasets were divided by protein sequence during 5-fold cross-validation (Figure S2). Thus, random splitting of these datasets in this manner during 5-fold cross-validation ensured no overlap in protein sequences between the training and validation sets. This sequence-based data splitting allowed us to test the generalization power of our ML models in predicting *T*_opt_, *k*_cat_/*K*_m,max_, the relative *k*_cat_/*K*_m_ vs *T* profile, and ultimately the temperature-dependent *k*_cat_/*K*_m_ for sequences that were not seen during training.

**Figure 2:**
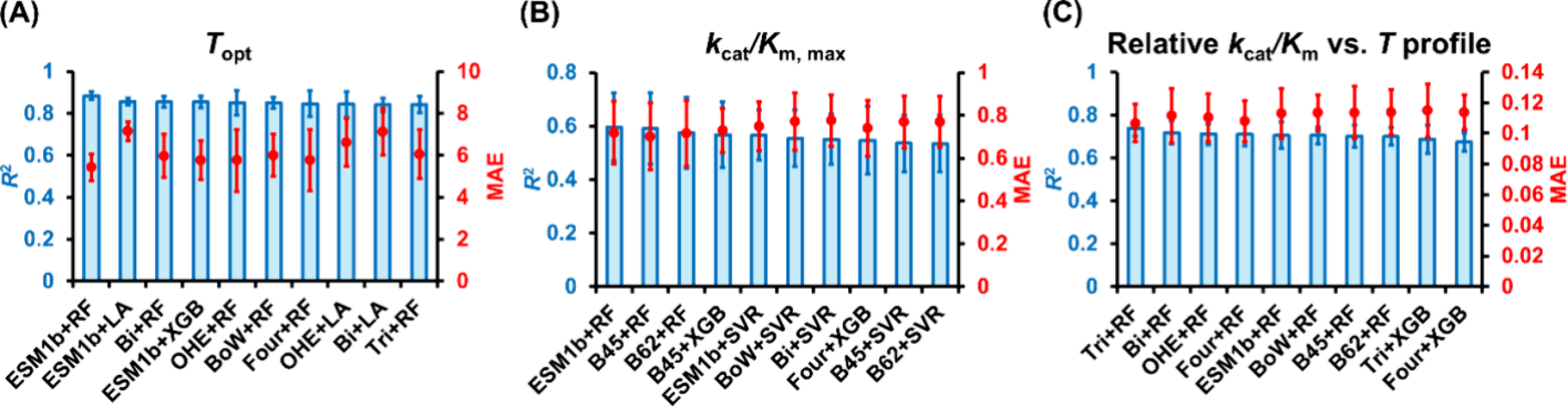
Evaluation of the prediction performance of various combinations of sequence representations and regression algorithms for (A) Module 1 (*T*_opt_), (B) Module 2 (*k*_cat_/*K*_m,max_), and (C) Module 3 (relative *k*_cat_/*K*_m_ vs *T* profile) using 5-fold cross-validation. The coefficient of determination (*R* ^2^; blue bars) and the mean absolute error (MAE; red circles) calculated by using the validation sets are shown as the evaluation metrics. For each module, the top ten combinations of sequence representation methods and regression algorithms, ranked by *R* ^2^ values, are presented. The *R* ^2^ values of the ten ML models shown above for training ranged from (A) 0.94 to 0.99 for *T*_opt_, (B) 0.63 to 0.99 for *k*_cat_/*K*_m,max_, and (C) 0.97 to 0.99 for the relative *k*_cat_/*K*_m_ vs *T* profile. The evaluation metric values shown here represent the average values from the 5-fold cross-validation. The error bars represent one standard deviation across the 5-fold cross-validation. The unit of MAE in (A) is °C, while it is dimensionless in (B) and (C). The sequence representation methods shown here include ESM1b, Bigram (Bi), One-Hot Encoder (OHE), Bag-of-Words (BoW), Fourgram (Four), Trigram (Tri), BLOSUM45 (B45), and BLOSUM62 (B62). The regression algorithms shown here include Random Forest (RF), LASSO (LA), Extreme Gradient Boosting (XGB), and Support Vector Regression (SVR).

Without any hyperparameter optimization, *R* ^2^ and MAE were calculated on the validation sets that contained no overlapping protein sequences with the training set for Modules 1, 2, and 3, and their averages and standard deviations were calculated over five folds. We did not perform hyperparameter optimization initially because the size of the entire dataset is relatively small. Previous studies showed that default hyperparameters can perform comparably to tuned ones, particularly when the tuning process is limited by a small dataset.^50,51^ Thus, default hyperparameters were used for each algorithm in our study. The accuracies of our top models for each module are notable when judged by the *R* ^2^ values calculated using the validation sets (Figure 2); *R* ^2^ for *T*_opt_ prediction = 0.84 – 0.88, *R* ^2^ for *k*_cat_/*K*_m,max_ prediction = 0.53 – 0.6, and *R* ^2^ for relative *k*_cat_/*K*_m_ vs *T* prediction = 0.67 – 0.74. Our *T*_opt_ models exhibited higher *R* ^2^ values than previously developed ML models for predicting *T*_opt_ from protein sequences, including both BGL and non-BGL sequences^21^ (referred to as broad-coverage models; *R* ^2^ = 0.5, when calculated on the validation set^52^). Our top ten *T*_opt_ models generally showed lower accuracy for BGL sequences with *T*_opt_ ≥ 50 °C (Figure S3A). This is possibly because of a slight bias in the training data distribution (*N* = 145 for *T*_opt_ < 50 °C and *N* = 115 for *T*_opt_ ≥ 50 °C in the whole dataset), difficulty in fully capturing the sequence characteristics of thermostable BGLs, and/or increased noise in high *T*_opt_ data (e.g., resulting from enzyme denaturation). The top five *k*_cat_/*K*_m,max_ models achieved *R* ^2^ ∼ 0.6, when measured on the validation sets. A fair comparison between our top *k*_cat_/*K*_m,max_ models and broad-coverage models (e.g., UniKP,^38^ EITLEM-Kinetics,^42^ and CataPro^43^), which predict a single representative, temperature-independent *k*_cat_/*K*_m_ value from a protein sequence and a substrate, is difficult due to differences in dataset scope: for example, the broad-coverage models considered non-BGL sequences and non-pNP-Glc substrates, unlike ours. Instead, our comparison between the top *k*_cat_/*K*_m,max_ models, and the broad-coverage models focused on BGL activity with pNP-Glc, using protein sequences from our BGL dataset. For the comparison, it was assumed that a representative, temperature-independent *k*_cat_/*K*_m_ value for a BGL sequence in the broad coverage models corresponds to the *k*_cat_/*K*_m,max_ value for the same sequence in our model. Notably, despite using a smaller dataset (*N* = 260), our top *k*_cat_/*K*_m,max_ models outperformed the broad-coverage *k*_cat_/*K*_m_ models (*N* = 910,^38^ 13 888,^42^ and 25 831 ^43^), at least for BGL activity with pNP-Glc: the broad-coverage models showed *R* ^2^ < 0.3 and MAE > 1.00 (Table 1), compared with *R* ^2^ ≥ 0.53 and MAE ≤ 0.78 for our *k*_cat_/*K*_m,max_ models (Table 1 and Figure 2B). The implication is that although some broad-coverage models achieved an *R* ^2^ of up to 0.68 across diverse enzymes and substrates,^38,42,43^ they exhibited relatively lower accuracy within BGL-specific sequence space with pNP-Glc. Our top ten *k*_cat_/*K*_m,max_ models generally perform better for BGL sequences with higher *k*_cat_/*K*_m,max_ values (Figure S3B) despite the slightly smaller dataset size for this group (*N* = 142 for *k*_cat_/*K*_m,max_ < 10^1.5^ mM^–1^ s^–1^ and *N* = 118 for *k*_cat_/*K*_m,max_ ≥ 10^1.5^ mM^–1^ s^–1^). This is possibly due to an improved signal-to-noise ratio within this group and/or a model bias toward higher *k*_cat_/*K*_m,max_ values. Our top models for each of the three modules consistently yielded strong performance during 5-fold cross-validation, with small to moderate standard deviations (Figure 2). The implication is that our model performance was not significantly dependent on the data splitting for training and validation in each fold. Among the various methods for numerical representation of protein sequences, ESM1b and *n*-gram encoding were commonly used in the top models across all three modules. The sequence similarity-based BLOSUM matrices were frequently utilized in the top models for Modules 2 and 3. Among various regression algorithms, tree-based ensemble methods, such as Random Forest (RF) and Extreme Gradient Boosting (XGB), generally achieved a strong predictive performance across models for all three modules. Apart from tree-based algorithms, several top models in Module 1 utilized LASSO (LA), while those in Module 2 employed Support Vector Regression (SVR). Our additional analysis showed that five ML models outperformed sequence similarity-based geometric averaging for *T*_opt_ prediction, while eight models demonstrated statistically superior performance for *k*_cat_/*K*_m,max_ prediction, avoiding potential data leakage (Supporting Information and Figure S4).

**Table 1:** Prediction Performance of Broad-Coverage Models on Representative, Temperature-Independent BGL *k*_cat_/*K*_m_ Values with pNP-Glc from Protein Sequences in the *k*_cat_ */K*_m,max_ Dataset, Compared with That of Our *k*_cat_ */K*_m,max_ Models^a^

		*R* ^2^	MAE
UniKP		0.051183	1.221249
EITLEM-Kinetics		0.194063	1.099578
CataPro		0.273163	1.042840
Our *k*_cat_/*K*_m,max_ model	ESM1b+RF	0.596786	0.718245
	ESM1b+SVR	0.565559	0.748908

a RF: Random Forest. SVR: Support Vector Regression.

### Evaluation of Model Performance across Various Sequence Characteristics

The top ML models for each of the three modules were further evaluated across various sequence characteristics. For this evaluation, the validation set was split into two subsets containing either wild-type or mutant sequences in each round of the sequence-based random data splitting. Generally, most models performed better for mutants than wild-type sequences (Figure 3A). Among others, the *k*_cat_/*K*_m,max_ prediction for wild-type sequences was the least accurate (Figure 3A). The low accuracy likely resulted from the limited wild-type sequence data (*N* = 119) relative to the vast wild-type sequence space for mapping *k*_cat_/*K*_m,max_. On the other hand, although the mutant dataset was of a comparable size (*N* = 141), the predictions for mutants were highly accurate, likely due to the locally dense distribution of the mutant sequence space within our dataset.

**Figure 3:**
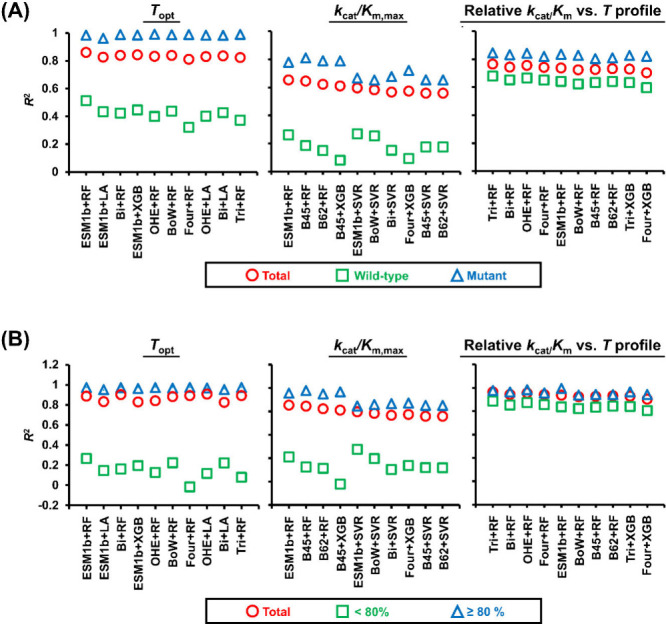
Prediction performance of the top ML models in each module on the validation subsets, created based on (A) wild-type versus mutant sequences and (B) maximum sequence identity. The *R* ^2^ value is presented as an evaluation metric, representing the average calculated from the validation sets across ten rounds of random data splitting by protein sequence. The sequence representation methods shown here include ESM1b, Bigram (Bi), One-Hot-Encoder (OHE), Bag-of-Words (BoW), Fourgram (Four), Trigram (Tri), BLOSUM45 (B45), and BLOSUM62 (B62). The regression algorithms shown here include Random Forest (RF), LASSO (LA), Extreme Gradient Boosting (XGB), and Support Vector Regression (SVR).

We also evaluated model performance using two different validation subsets split according to maximum sequence identity (SI) relative to the training set in each round of sequence-based random data splitting. The maximum SI of a protein sequence in the validation set was determined by the highest value among its pairwise sequence identities calculated with all protein sequences in the training dataset. Due to the sequence-based data splitting, the maximum SI of a protein sequence in the validation set is always less than 100%. Given that the maximum SIs of wild-type sequences are usually lower compared to mutant sequences, similar model performance was observed (Figure 3B): the prediction performance of the models was generally stronger when the maximum SIs between the target sequences in the validation set and the sequences in the training set were high (>80%), as observed with previous ML models for *k*_cat_.^38,39^

### Construction of the Three-Module ML Frameworks and Their Performance Comparison with the Single-Module ML Frameworks

The top 20 ML models in each of the three modules were combined for prediction of *k*_cat_/*K*_m_ values and their temperature dependency from protein sequences. It should be noted that the ML models were trained individually as above and then integrated under the three-module framework. For the validation of the entire three-module ML framework, *T*_opt_ and *k*_cat_/*K*_m,max_ values for a protein sequence of interest were predicted by ML models in Modules 1 and 2, respectively, and then fed to the ML models in Module 3 to calculate *k*_cat_/*K*_m_ values at other temperatures. The various ML model combinations were evaluated based on their *R* ^2^ values for predicting *k*_cat_/*K*_m_ values at designated temperatures, calculated on the validation set in each round of the sequence-based random data splitting. The *R* ^2^ values for the 8000 combinations ranged from 0.106 to 0.377 (Table S3), with the top ten combinations achieving *R* ^2^ values of ≥0.375 (Figure 4A). All the top ten combinations outperformed the sequence similarity-based geometric averaging calculations (Figure 4A). Interestingly, while some of the top ten combinations included ML models that also ranked in the top ten individually, the remaining combinations utilized some ML models that did not rank in the top ten individually (Figures 2, S4, and 4A). This is presumably due to the complex interplay among the three modules in protein-sequence- and temperature-dependent *k*_cat_/*K*_m_ prediction within the combined framework. Notably, ESM1b+SVR was exclusively used for the second module in all the top ten combinations (Figure 4A). This is likely due to its robust predictive performance, as it exhibited the smallest difference between *R* ^2^ values for training (=0.703) and validation (=0.566) among the top five *k*_cat_/*K*_m,max_ models ranked individually (Figures 2 and S4). In contrast, other sequence representation methods and regression algorithms were frequently used in the first and third modules among the top ten combinations. This suggests that our three-module framework enables model optimization within each module without being restricted to specific protein sequence representation methods or regression algorithms.

**Figure 4:**
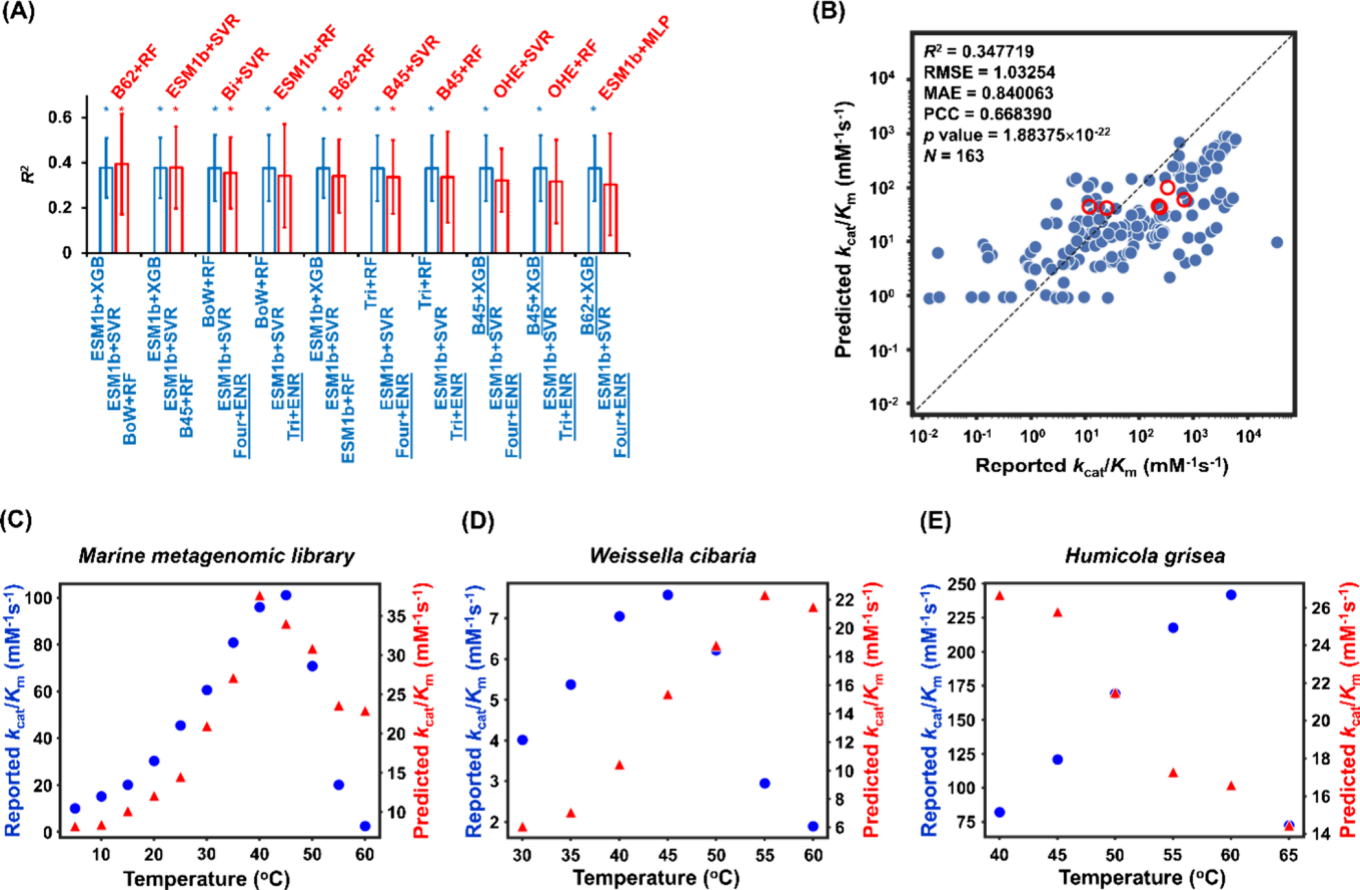
(A) The *R* ^**2**^ values of the top ten ML model combinations within the three-module framework (blue) and the top ten ML models in the single-module framework (red). (B) Correlation between predicted and reported *k*_cat_/*K*_m_ values across various protein sequences and temperatures, and (C–E) comparison of predicted (red) and reported (blue) *k*_cat_/*K*_m_ values as a function of protein sequence and temperature. In (A), the labels of the ML models in Modules 1, 2, and 3 within the three-module framework (blue) are displayed from the top left to the bottom right, respectively. The ML models that did not rank in the top ten individually are underlined. The *R* ^2^ values shown in (A) represent the averages calculated from the validation sets across ten rounds of random data splitting by protein sequence, with error bars indicating one standard deviation. In (A), *: *p* < 0.05 from a two-sided Wilcoxon signed-rank test, compared to the sequence similarity-based geometric averaging calculations. In (B)–(E), ESM1b+XGB for Module 1, ESM1b+SVR for Module 2, and BoW+RF for Module 3 were used. For the illustrations in (B)–(E), the most representative validation dataset from the ten rounds of data splitting was selected, based on its *R* ^2^ value being closest to the average *R* ^2^ value calculated from the validation sets across the ten rounds. In (B), the RMSE, MAE, PCC, and *p* values were calculated from the most representative validation dataset. In (B), the red circles represent data for the two ancestral sequences. The sequence representation methods shown here include ESM1b, Bag-of-Words (BoW), BLOSUM45 (B45), Fourgram (Four), Trigram (Tri), BLOSUM62 (B62), Bigram (Bi), and OHE (One-Hot-Encoder). The regression algorithms shown here include Extreme Gradient Boosting (XGB), Support Vector Regression (SVR), Elastic Net Regression (ENR), Random Forest (RF), and Multilayer Perceptron (MLP).

To evaluate the merits of the three-module ML framework, single-module ML models were constructed as a control using the same protein sequence representations, regression algorithms, and dataset. These single-module ML models were designed to learn and predict protein sequence- and temperature-dependent *k*_cat_/*K*_m_ values directly from the training data, without deconvoluting the three related aspects (i.e., *T*_opt_, *k*_cat_/*K*_m max_, and the relative *k*_cat_/*K*_m_ vs *T* profile). The *R* ^2^ values of the top ten single-module ML models ranged from 0.303 to 0.394, calculated on the validation set in each round of the sequence-based random data splitting (Figure 4A). It should be noted that the prediction performance of the top ten single-module ML models depended on the data splitting more significantly than the top ten three-module ML combinations, when judged by the standard deviation of *R* ^2^ values (Figure 4A). Of the ten single-module ML models, only five outperformed the sequence similarity-based geometric averaging calculations (Figure 4A). Among others, the top two three-module ML frameworks and the top three single-module ML models, each with similar evaluation metric values (*R* ^2^, PCC, MAE, and root mean square error (RMSE)) for the validation sets, were selected for further comparison. Notably, the single-module models exhibited greater discrepancies in these metrics between training and validation (Tables S4 and S5). These comparisons highlight the strong potential of our three-module ML architecture in reducing data split dependence and mitigating the overfitting observed in single-module models. Additionally, when evaluated on subsets of the validation dataset, the top two three-module ML frameworks generally showed higher prediction accuracy for low-similarity sequences compared to the top three single-module ML models, as judged by the four evaluation metrics (Tables S6 and S7). The implication is that the three-module architecture may facilitate more effective navigation of the distant sequence space formed by BGLs with relatively low sequence similarity.

### Protein Sequence- and Temperature-Dependent *k*_cat_/*K*_m_ Prediction by the Three-Module ML Frameworks

The top two three-module combinations were characterized further for their protein sequence- and temperature-dependent *k*_cat_/*K*_m_ predictions. The accuracy of the top two three-module ML frameworks is notable (Table S4), considering that the RMSE of experimental log *k*_cat_/*K*_m_ values is generally around 1.^53^ When the predicted values from the top two combinations were compared with the reported values, the *k*_cat_/*K*_m_ predictions were found to be reasonable, spanning several orders of magnitude across a range of protein sequences and temperatures (Figures 4B and S5). The prediction errors were generally higher at both low and high reported *k*_cat_/*K*_m_ values for the top two combinations (Figures 4B and S5), presumably due to the limited data available in these categories. Additionally, the observed trend of overestimating lower *k*_cat_/*K*_m_ values and underestimating higher ones may result from regression dilution, a common statistical effect in which noise in the input features flattens the slope of the correlation between predicted and true target values.^54^ To explore whether prediction accuracy could be further improved, hyperparameter tuning was performed on the ML models in the top two three-module combinations. Although tuning may help build individual models with minimal overfitting, it led to only modest or no improvement in the performance of the individual modules as well as in the overall combined framework (Supporting Information and Figure S6).

For some BGL sequences, the *k*_cat_/*K*_m_ vs temperature profiles generated from the top two three-module ML frameworks were largely in good agreement with those reported (Figures 4C, S7, and S8). For certain BGL sequences, the predicted *k*_cat_/*K*_m_ vs temperature profiles were notably shifted compared to the reported profiles, despite their similar shapes and comparable *k*_cat_/*K*_m_ values (Figures 4D, S7, and S8). This discrepancy is primarily due to inaccuracies in the *T*_opt_ predictions by the ML model in Module 1. In some cases, although the predicted and reported profiles had similar shapes, the magnitudes of the *k*_cat_/*K*_m_ values differed substantially (Figures S7 and S8). In other cases, the optimum temperatures, profile shapes, and magnitudes of the *k*_cat_/*K*_m_ values all differed substantially (Figures 4E, S7, and S8). This discrepancy is likely due to error propagation across the three models within the three-module framework. It should also be noted that model combinations should be carefully evaluated for selection rather than relying solely on evaluation metrics (e.g., *R* ^2^). For example, the top third and fourth combinations (i.e., BoW+RF for Module 1, ESM1b+SVR for Module 2, and Four or Tri+ENR for Module 3) predicted the relationship between *k*_cat_/*K*_m_ and temperature linearly, failing to capture the nonlinear trend (Figures S9 and S10). In these combinations, where errors occurred in the prediction of *k*_cat_/*K*_m,max_ from ESM1b+SVR in Module 2, the ENR algorithm in Module 3 produced negative linear trends in relative activity for the overall fitting of *k*_cat_/*K*_m_ values.

### Testing of the Three-Module ML Frameworks with Ancestral BGL Sequences

To further evaluate whether our three-module ML frameworks generalize well to unseen sequences beyond our original dataset, we tested the three-module ML frameworks using BGL sequences that have not been identified. To generate novel sequences that are likely to possess BGL activity and share some similarity with the BGL sequences in the original dataset, we utilized phylogenetic analysis. Among various BGLs, we constructed a phylogenetic tree involving a highly stable and well-expressed BGL from *Pyrococcus furiosus* (PfBGL)^55^ (Figure S11). This is because this protein can tolerate multiple potentially destabilizing mutations,^56,57^ which increases the likelihood of generating functional ancestral sequences that are both well-folded and highly expressible. Among several ancestral sequences identified by this analysis, those at Nodes 13 and 15 were selected for further characterization (Figure S11). This is because these two ancestral sequences contain the “NEP” and “TENG” sequence motifs (Figure S12), which are known to be highly conserved among many BGLs.^47^ Moreover, these two ancestral sequences have a pairwise sequence identity (SI) of ∼ 80–90% with PfBGL (Table S8), a range where our ML model predictions were relatively accurate (see Figure 3B and Table S6). The two ancestral sequences also exhibit a similar SI to *Thermococcus pacificus* β-glucosidase (TpBGL) (Table S8), which was also included in our dataset (Table S1). Compared to PfBGL and TpBGL, these ancestral sequences contain ∼60–80 mutations, which are much higher than the numbers typically introduced during conventional mutagenesis experiments. The two ancestral sequences were expressed in *Escherichia coli*, purified using a His-tag, and their enzyme activity (*k*_cat_/*K*_m_) was measured with pNP-Glc at three different temperatures. Notably, the top two three-module ML frameworks described above, which were trained on the original dataset not containing any ancestral sequences, predicted the *k*_cat_/*K*_m_ values of the two ancestral sequences in a temperature dependent manner with reasonable errors (i.e., within an order of magnitude; see red circles in Figures 4B and S5). Thus, our three-module ML framework demonstrates the generalization power beyond the BGL sequences in the original dataset.

## Discussion

In this study, we constructed a unique three-module ML framework that collectively predicts β-glucosidase *k*_cat_/*K*_m_ values as target regression variables based on the protein sequence and temperature. Each module in the framework was designed to capture different aspects of the interplay among protein sequence, temperature, and *k*_cat_/*K*_m_ for β-glucosidase activity. The models within these modules work in concert to predict protein-sequence- and temperature-dependent *k*_cat_/*K*_m_ values, focusing on the functional sequence space for β-glucosidase activity. The modular structure of this framework allows for the optimization of ML models within each of the three modules, collectively offering notable generalization performance in predicting temperature-dependent *k*_cat_/*K*_m_ values for BGL sequences that are unseen during training. No existing ML model predicts *k*_cat_/*K*_m_ as a nonlinear function of temperature, making our approach unique. Our results also highlight the advantages of the three-module ML framework over the conventional single-module approach, particularly in mitigating prediction performance variability due to data splitting and reducing overfitting.

We anticipate that our multimodule ML framework approach can be directly applied to other enzyme systems, facilitating comprehensive functional annotation of proteins for various applications. However, the generalizability of the three-module ML framework depends on several key factors, including the availability of diverse sequence–*k*_cat_/*K*_m_ datasets on a common substrate and *k*_cat_/*K*_m_ data across a broad temperature range. Additional considerations, such as the presence or absence of cofactors or the influence of pH, may also be necessary if enzyme activity is strongly dependent on these biochemical factors. When successfully applied to other protein systems (particularly those where ML models in the top-performing combinations use sequence representations with physically meaningful or biologically interpretable features), feature importance or SHapley Additive exPlanations (SHAP) analysis may offer valuable insights into the key features underlying various aspects of enzymatic properties. Unfortunately, this was not feasible in our current study because the models in the top-performing three-module combinations generally used ESM-1b or BoW in Modules 1 and 2. These representations lack physical or biological interpretability: ESM-1b embeddings are abstract, and BoW ignores context and position.^58,59^ Although alternative representations were used in Module 3 of the top-performing combinations, its output is the relative *k*_cat_/*K*_m_–temperature profile shape, which also limits the usefulness of feature importance or SHAP analysis. Beyond enzyme systems, a similar multi-module ML framework approach could be applied for other types of data involving temporal or spatial progression, where one module could predict initial data while another could model its temporal or spatial evolution.

An ML model for accurately predicting *k*_cat_/*K*_m_ values is very useful, as this ratio serves as a measure of catalytic efficiency for direct comparisons across different enzymes. *K*_*m*_ is often considered an indicator of the binding affinity between an enzyme and its substrate and might be determined from theoretical thermodynamic calculations by using enzyme and substrate structures for their binding. While this interpretation is acceptable in some cases, it is not valid for many other enzymes, for example, which form multiple complexes with substrates, both noncovalently and covalently. A similar complication is realized with *k*_cat_; *k*_cat_ is a combination of multiple rate constants when a covalent enzyme–substrate complex is formed during catalysis. A previous machine learning model has demonstrated the ability to predict *k*_cat_ values using active site information, metabolite concentrations, and metabolic flux calculations;^40^ however, such data are often available only for a limited number of enzymes.^36,40^ While conformational dynamics around the active site provide valuable insights into enzyme activity, computational tools that directly capture the relationships between conformations and the chemical steps of catalysis remain scarce.^25^ For the previous ML models for *k*_cat_, *K*_m_, and *k*_cat_/*K*_m_, the prediction of *k*_cat_/*K*_m_ is less accurate than *k*_cat_ or *K*_m_ alone.^38,42^ Even when *k*_cat_ and *K*_m_ are individually predicted with reasonable accuracy, the errors are propagated when their ratio is taken to compute the *k*_cat_/*K*_m_ value.^38^ Therefore, an ML model with high prediction accuracy for *k*_cat_/*K*_m_ from the protein sequence as an input would be highly valuable. Importantly, *k*_cat_/*K*_m_ is a function of environmental factors as well; for example, temperature itself can vary *k*_cat_/*K*_m_ by several orders of magnitudes. While empirical rules, such as the Q10 rule, can estimate the increasing trend of *k*_cat_/*K*_*m*_ with rising temperature to some extent,^5^ they do not fully capture the complete *k*_cat_/*K*_m_ vs *T* profile. Instead, this profile is shaped by a complex balance of kinetic and thermodynamic factors.^52,60,61^ Unfortunately, accurately predicting the temperature dependence of a standardized enzyme activity parameter remains highly challenging: the EF-UniKP model achieved an *R* ^2^ value of 0.31 for temperature-dependent *k*_cat_ predictions on a validation set, in which either a protein sequence or a substrate did not overlap with those in the training set.^38^ Moreover, it is unclear whether the EF-UniKP model can predict nonlinear activity–temperature profiles around the optimal temperature. In contrast, our top two ML model combinations within the three-module framework achieved an *R* ^2^ value of ∼0.38 on validation sets containing protein sequences not seen during training and can generate the *k*_cat_/*K*_*m*_ profiles as a function of the temperature for a BGL sequence of interest. Unfortunately, direct comparisons between the EF-UniKP model and our ML framework are difficult due to differences in data nature and scope (e.g., for BGL-specific *k*_cat_/*K*_m_ vs broad range *k*_cat_ predictions). Nevertheless, we anticipate that our ML framework would likely outperform other models in quantitatively predicting enzyme activity from protein sequence and temperature, at least for BGL activity with pNP-Glc. This expectation is based on our finding that these models produced relatively inaccurate predictions of BGL *k*_cat_/*K*_m_ values with pNP-Glc, and on the observation that incorporating temperature as an additional feature, beyond sequence, further complicates prediction.^38^ Overall, our results suggest that the three-module ML framework could serve as a facile platform for predicting the enzyme activity and its temperature dependence.

Interestingly, despite using a smaller dataset, our top individual ML models, when evaluated independently of the three-module framework, appeared to achieve evaluation metrics that were higher than previous ML models. For example, our top ML models for predicting *T*_opt_ (*N* = 260) achieved an *R* ^2^ of 0.84–0.88 calculated from the validation sets. In comparison, the broad-coverage models (*N* > 2600), designed to predict *T*_opt_ for any protein sequences,^21^ showed an *R* ^2^ = 0.5 on the validation set.^52^ Our top *k*_cat_/*K*_*m*, max_ models (*N* = 260) achieved *R* ^2^ values of ∼0.6 on the validation sets, outperforming the broad-coverage *k*_cat_/*K*_m_ models (*N* ≥ 910) with *R* ^2^ < 0.3, at least for BGL activity with pNP-Glc. As noted earlier, a fair comparison between our *T*_opt_ and *k*_cat_/*K*_m,max_ models, and the broad-coverage models is challenging. This is because our models were built on a smaller, specific subset of protein sequences known to exhibit BGL activity with pNP-Glc, whereas the broad-coverage ML models were trained on larger datasets containing a wide variety of proteins and substrates curated in public databases.^37,38,41,42^ Importantly, in these public databases, only a small amount of catalytic data is available for specific enzyme groups, which may limit machine learning in predicting specific enzyme activity. For example, while BRENDA contains data on 8475 different EC numbers, the average number of data entries per EC number is only 10.2 (=86517/8475) for *k*_cat_, 20.2 (=170819/8475) for *K*_*m*_, and 4.6 (=39008/8475) for *k*_cat_/*K*_*m*_, as of March 2025. These numbers decrease further when a specific substrate is selected. In contrast, our BGL dataset contains 885 entries with pNP-Glc as a substrate, including 260 unique BGL sequences, curated from multiple sources. Thus, our dataset offers a more focused resource, facilitating machine learning within a specific functional sequence space. The implication is that prediction accuracy could be improved by adjusting coverage, as evidenced by the well-documented trade-off between accuracy and coverage in machine learning.^62,63^

Predictive models that perform well on enzyme sequences absent from the training dataset must have learned generalizable information encoded in latent spaces. To assess this generalizability, we split the data by protein sequence for training and validation, ensuring that no data entries with the same protein sequence appeared in both sets. This approach is particularly crucial for evaluating the combined three-module framework’s ability to predict temperature-dependent *k*_cat_/*K*_m_ values for a protein sequence of interest. In sequence-blinded data splitting, data entries for the same amino acid sequence at different temperatures may be distributed across both the training and the validation sets. When *k*_cat_/*K*_m_ data for a given sequence at a specific temperature is included in the training set, predicting *k*_cat_/*K*_m_ values at other temperatures for the same sequence becomes relatively straightforward. In contrast, data splitting by protein sequence prevents such overlaps, allowing for a rigorous assessment of the model’s predictive performance. Our ML models and their combinations demonstrated reasonable predictive power for *k*_cat_/*K*_m_, not only for BGL sequences excluded from training but also for ancestral protein sequences generated through phylogenetic analysis. This suggests that our ML models and their combinations successfully captured, at least to some extent, generalizable protein sequence attributes that determine the catalytic activity of BGLs in a temperature dependent manner.

Sequence–function–temperature relationships are highly convoluted, making it challenging to develop a computational tool for accurate prediction. While our three-module ML framework demonstrates non-negligible predictive power, further improvements are needed to enhance its accuracy. For example, both our individual ML models and their combinations within the three-module framework exhibited considerably weaker predictive power for wild-type sequences compared with mutant sequences. The inclusion of BGL mutant data in our dataset helped our ML models capture the characteristics of specific regions within narrow sequence spaces. Unfortunately, learning the local sequence space does not necessarily contribute to learning the broader, nonlocal BGL sequence space.^64^ A similar limitation in learning the wild-type sequence space was also observed in the previous ML models for *k*_cat_ prediction.^37,38^ Given the relatively lower degree of sequence similarity among wild-type BGL sequences compared to mutant BGL sequences, the sequence space occupied by wild-type BGLs is significantly larger than that of mutant BGLs. However, the ability of our ML models to learn the wild-type BGL sequence space is limited by the small number of wild-type data, which are just comparable in size to the mutant dataset. It should also be noted that the predictive power of our three-module ML framework is generally weaker for both high and low *k*_cat_/*K*_m_ values, presumably due to the smaller amount of data in these ranges. The development of more precise ML models and frameworks will undoubtedly benefit from a larger dataset encompassing wild-type BGL enzymes and their mutants across a diverse sequence and activity space. Functional characterizations under various conditions in genomic and metagenomic studies will be especially valuable as these efforts are likely to uncover novel and uncharacterized BGL sequences that may encode catalytic activities. Additionally, incorporating negative data (a set of protein sequences that exhibit no BGL activity) into the learning process could further improve machine learning of the functional landscape and the detection of higher-order relationships across sequences. Several computational strategies could also be considered to expand the effective dataset size. For example, data augmentation through *in silico* labeling of protein sequences using catalytic information could help enrich the training set. Semisupervised learning methods, which may leverage both labeled *k*_cat_/*K*_m_ data and unlabeled protein sequence data, could also enhance learning from the limited labeled data. Transfer learning from related datasets may improve generalization without requiring large-scale labeled data. Collectively, these efforts will extend the boundaries of knowledge in functional sequence space, ultimately aiding in the development of more accurate ML models and frameworks for precise sequence-function-temperature mapping.

To further improve the prediction accuracy, it is worth considering the evaluation of newly developed protein sequence representation methods. Our ML models in the top-performing three-module combinations, particularly in Modules 1 and 2, utilized ESM-1b. Therefore, ESM-2 is of particular interest, as it has been shown to outperform ESM-1b and other pretrained protein language models through improvements in architecture, training strategies, and the scale of training data.^65^ A more systematic evaluation of different sequence representation methods (such as those benchmarked in the Tasks Assessing Protein Embeddings (TAPE)) could help identify the most promising approaches for enhancing predictive performance.^66^ In addition, incorporating features beyond protein sequences may also be beneficial. For example, structural features derived from contact maps of protein 3D structures enhanced *k*_cat_ prediction in DeepEnzyme, particularly for enzymes with low sequence similarity,^67^ although similar considerations have not consistently improved accuracy in other studies.^41^ Refining regression algorithms (such as modifying ensemble strategies in gradient boosting, as done in Enzyme Catalytic Efficiency Prediction) may also contribute to better performance.^68^ More sophisticated learning approaches, such as deep neural networks, are effective at capturing complex nonlinear patterns but were not feasible in our study due to the risk of overfitting with the small sample size. Instead, we employed simpler models with fewer parameters, which are better suited for limited datasets. However, as more *k*_cat_/*K*_m_ data become available, these advanced model architectures may be explored to further enhance the prediction accuracy.

## Materials and Methods

### Dataset Preparation

Catalytic efficiencies (*k*_cat_/*K*_m_) of β-glucosidases and temperatures at which their *k*_cat_/*K*_m_ values were measured were manually compiled from relevant sources with protein sequence information extracted from reported accession numbers through GenBank and UniProt databases. The *k*_cat_/*K*_m_ values and assay temperatures archived in BRENDA were reviewed and updated with the linked references as necessary. Some literature reports a *k*_cat_/*K*_m_ value at a specific temperature, along with enzyme activity data at other temperatures measured at a fixed substrate concentration. In such cases, *k*_cat_/*K*_m_ values at the additional temperatures were estimated using the reported reference *k*_cat_/*K*_m_ value and the activity–temperature relationship measured at the fixed substrate concentration. These estimated values were included in our dataset. To establish an approximate normal distribution, log_10_ transformations were applied to the *k*_cat_/*K*_m_ values. To minimize noise and errors in the data and improve model performance, outliers in the collected *k*_cat_/*K*_m_ values were removed based on the interquartile range of the datasets, as described elsewhere.^39^ Consequently, data points with *k*_cat_/*K*_m_ values of <10^–2^ and >10^5^ mM^–1^ s^–1^ were excluded, as these values fall outside the typical range for most enzymes.^44^

From the entire dataset constructed in this manner, a subset was created by selecting each protein sequence and its corresponding temperature at which the highest *k*_cat_/*K*_m_ value was observed. This subset was used to construct and validate ML models for predicting the optimal temperature (*T*_opt_) from protein sequences. Similarly, another subset of the entire dataset was generated by selecting each protein sequence along with the highest *k*_cat_/*K*_m_ value at its corresponding *T*_opt_. This subset was used for the construction and validation of the ML models for predicting the maximum *k*_cat_/*K*_m_ (*k*_cat_/*K*_m,max_) from protein sequences. For a protein sequence with a single *k*_cat_/*K*_m_ value measured at a specific temperature, these values were directly assigned as *k*_cat_/*K*_m,max_ and *T*_opt_, respectively, for the sequence. Lastly, the entire dataset was transformed by normalizing *k*_cat_/*K*_m_ values by *k*_cat_/*K*_m,max_ for each protein sequence at temperatures relative to its *T*_opt_. The resulting dataset was used to develop and validate ML models for predicting relative *k*_cat_/*K*_m_ vs *T* profiles from protein sequences and temperatures.

### Enzyme Sequence Representation

Various methods were employed to represent protein sequences numerically, including ESM1b, one-hot-encoding, bag of words, *n*-gram (a string of *n* consecutive amino acids; *n* = 2, 3, and 4), and BLOSUM scoring matrices (BLOSUM45 and BLOSUM62). For protein sequence representations using BLOSUM scoring matrices, amino acid sequences were aligned using a multiple sequence alignment tool, CustalW.^69^

### Regression Algorithms

Various regression algorithms were utilized during the ML model constructions, such as linear regression, the Least Absolute Shrinkage and Selection Operator (LASSO), decision tree, Random Forest (RF), Support Vector Regression (SVR), Multi-Layer Perceptron (MLP), Elastic Net Regression (ENR), and Extreme Gradient Boosting regression (XGB).

### 5-Fold Cross-Validation

The ML models were constructed by combining each enzyme sequence representation method with various regression algorithms. Their performance and robustness were evaluated through 5-fold cross-validation on the entire dataset, with 80% of the data entries selected as the training set and the remaining 20% as the validation set. During data splitting, entries were divided by protein sequences to ensure that there were no protein sequence overlaps between the training and validation sets. For each ML model, the average *R* ^2^ and MAE values were calculated across five validation sets, one for each fold.

### Model Training and Validation during 10 Rounds of Random Sequence-Based Data Splitting

To thoroughly evaluate the performance of selected ML models and their combinations, ten rounds of random data splitting for training and validation were performed. In each round, the entire dataset was randomly partitioned into five subsets based on protein sequences, with 80% used for training and the remaining 20% for validation. This data splitting process was independent of the 5-fold cross-validation while ensuring that no identical protein sequences were present in both the training and validation sets. For model performance evaluation using a subset of validation sets based on sequence identity, sequence identity was calculated by aligning each pair of sequences with the pairwise2 module from the Biopython library.

### Statistical Analysis

A two-sided Wilcoxon signed-rank test was employed to examine if the differences in performance between the ML models and frameworks, and the sequence similarity-based geometric averaging calculations were statistically significant. A correlation between the reported and predicted values was statistically examined by calculating the *p* values and Pearson correlation coefficient (PCC) using the SciPy library in Python.^70^

### Benchmarking UniKP, EITLEM-Kinetics, and CataPro on the *k*_cat_/*K*_m,max_ Datasets

We benchmarked our *k*_cat_ */K*_m, max_ predictions against UniKP,^38^ EITLEM-Kinetics,^42^ and CataPro^43^ using our BGL dataset. Representative, temperature-independent BGL *k*_cat_/*K*_m_ values were obtained by inputting protein sequences from our *k*_cat_ */K*_m,max_ dataset into these models, using pNP-Glc as the substrate. For UniKP, the models were downloaded from the official GitHub repository (https://github.com/Luo-SynBioLab/UniKP) and executed with the default settings provided by the authors. EITLEM-Kinetics was run through its publicly available Python package (https://github.com/XvesS/EITLEM-Kinetics) with default hyperparameters and without additional optimization. CataPro predictions were obtained using the command-line tool available at https://github.com/zchwang/CataPro, with the standard configuration and no parameter tuning. All models were run in accordance with the protocols recommended in their respective GitHub repositories. The performance of UniKP, EITLEM-Kinetics, and CataPro was evaluated by using *R* ^2^ and MAE between reported and predicted values across all 260 BGL sequences.

## Supplementary Material





## Data Availability

The datasets, scripts, and code for the ML models and their combinations described in this study are publicly available at https://github.com/emreerkanli/BGL-kcatKm-predictor.

## Notes

Data on β-glucosidases (XLSX)
Descriptions of data collection, sequence similarity-based geometric averaging calculations, hyperparameter tuning, experimental reagents, ancestral sequence reconstruction, the expression and purification of *Pyrococcus furiosus* and *Thermoanaerobacterium aotearoense* β-glucosidase mutants, enzyme assays, and other tables and figures (PDF)

## Abbreviations

BGL: β-glucosidase
Bi: bigram
BLOSUM: blocks substitution matrix
BoW: bag-of-words
B45: BLOSUM45
B62: BLOSUM62
ENR: elastic net regression
Four: fourgram
*k*_cat_/*K*_m,max_: *k*_cat_/*K*_m_ at *T*_opt_
*k*_cat_/*K*_m_ vs *T*: *k*_cat_/*K*_m_ vs temperature
LA: LASSO
LASSO: least absolute shrinkage and selection operator
MAE: mean absolute error
ML: machine learning
MLP: multilayer perceptron
OHE: one-hot-encoder
PCC: Pearson correlation coefficient
PfBGL: *Pyrococcus furiosus* β-glucosidase
pNP-Glc: *p*-nitrophenyl β-d-glucopyranoside
RF: random forest
RMSE: root-mean-square error
*R* ^2^: coefficient of determination
SI: sequence identity
SVR: support vector regression
*T*_opt_: optimum temperature
TpBGL: *Thermococcus pacificus* β-glucosidase
Tri: trigram
XGB: extreme gradient boosting
