# Supplementary material for: A Three-Module Machine Learning Framework for Protein Sequence- and Temperature-Dependent k cat/K m Prediction in β‑Glucosidases
Source: ACS Synth Biol. 2025 Oct 2;14(10):3927–39. doi: 10.1021/acssynbio.5c00257 (PMC12538591; doi:10.1021/acssynbio.5c00257)
Supplement: Supplementary file 2 [file sb5c00257_si_002.pdf]

Supporting Information

A Three-Module Machine Learning Framework for Protein Sequence- and Temperature-Dependent k_{cat}/K_m Prediction in β-Glucosidases

Mehmet Emre Erkanli, Yunseok Jang, Ali Malli, Khalid El-Halabi, Chaehyun Ryu, Jin Ryoun Kim

Data collection and curation of k_{cat}/K_m values for β-glucosidases

An initial search in September 2022-December 2024 through BRENDA[1] returned 331 entries on k_{cat}/K_m values measured at various temperatures for BGLs, of which the entries measured using pNP-Glc were the most common, totaling 86 entries. To minimize noise in datasets due to errors created during the transfer of k_{cat}/K_m values to BRENDA from literature, the references associated with the entries in BRENDA were examined and the information on k_{cat}/K_m was updated, as needed. To supplement the relatively small number of entries obtained through the BRENDA search, a manual PubMed search was conducted, yielding several hundred additional entries. When gathering data, both wild-type BGLs and their mutants—whether naturally occurring or engineered—were considered, and their amino acid sequences, k_{cat}/K_m values, and assay temperatures were collected. We also included our own experimental data (N=23) on k_{cat}/K_m values measured with BGLs from *Pyrococcus furiosus* (PfBGL) and *Thermoanaerobacterium aotearoense* (TaBGL) as well as their point mutants generated from our other studies, both published[2] and unpublished. The collected BGL sequences were highly diverse, containing 119 wild-types and 141 mutants that are derived from 96 species covering most biological kingdoms: the average pair-wise sequence identity of the collected BGLs is 0.34±0.22, with an average sequence length of 523±133 amino acids (**Table S2**). The final dataset included 260 unique protein sequence entries and 885 total data entries, covering k_{cat}/K_m values spanning 6 orders of magnitude and temperatures from 4 to 105 °C (**Figure S1**). The k_{cat}/K_m data followed an approximate normal distribution on a log scale (**Figure S1A**).

Comparison of ML models with sequence similarity-based geometric averaging calculations

Since our whole dataset contains protein sequences that are relatively similar due to shared BGL activity and the inclusion of point mutants of wild-types, one may approximate the T_{opt} value of a protein sequence in a validation set simply from the T_{opt} values of the most similar sequences in a training set. This is known as a data leakage issue.[3-5] Thus, we compared the prediction performance of the top models for T_{opt} with the geometric averaging of these values, calculated over the three most similar BGL sequences in the training set. For T_{opt} prediction, five ML models provided results that are statistically better than those obtained by sequence similarity-based geometric averaging (**Figure S4A**). When the data leakage was examined similarly for $k_{cat}/K_{m, max}$ prediction, eight ML models showed statistically superior performance compared to the geometric averaging calculation (**Figure S4B**). No similar analysis was conducted for models in Module 3, as no significant data leakage issues were anticipated when predicting relative k_{cat}/K_m versus T profiles. This is because the relative enzyme activity vs. temperature profiles are generally not highly conserved among similar protein sequences.[6, 7]

Hyperparameter tuning

We performed hyperparameter tuning on the ML models within each of the three modules in the top two performing three-module combinations.

For Module 1, we performed hyperparameter optimization of the XGBoost regressor using Bayesian optimization framework implemented via Optuna.[8] Key hyperparameters targeted for optimization included those related to booster type (either *gbtree* or *dart*), regularization (lambda, alpha, and gamma), minimum loss reduction (gamma), tree complexity (max_depth and min_child_weight), and sampling (subsample and colsample_bytree). The learning rate (eta) was also included in the search. For the *dart* booster, additional dropout-specific parameters (sample_type, normalize_type, rate_drop, and skip_drop) were optimized. To mitigate overfitting, we incorporated early stopping (after 10 rounds without improvement) and evaluated model performance using repeated k-fold cross-validation (5 folds, 2 repeats). This tuning approach led to a modest improvement in the accuracy of Module 1, with the R^2 value increasing from 0.82870 to 0.87350 across ten rounds of random data splitting by protein sequence. When similar hyperparameter optimization was applied to Modules 2 and 3 using Bayesian optimization via Optuna,[8] the efforts yielded no notable performance improvement, as indicated by an R^2 increase of < 0.002. When the hyperparameter-tuned model was used for Module 1, the overall improvement in R^2 within the combined three-module framework was not substantial: the R^2 value across ten rounds of random data splitting by protein sequence increased by less than 0.0002.

To assess model generalization, we analyzed the training and validation RMSE loss curves across boosting rounds for the model in Module 1 (**Figure S6**). The model evaluation employed a repeated k-fold cross-validation strategy (5 folds, 2 repeats) to robustly estimate generalization performance. In each fold, the model was trained for up to 100 boosting rounds, with early stopping enabled after 10 rounds without improvement. The loss curves (**Figure S6**) showed that the validation error plateaued without diverging from the training loss, suggesting that the model did not overfit. The small gap between training and validation RMSEs may be attributed to the rounding of target values in the dataset, as most optimum temperatures are reported as rounded figures (e.g., 40, 50, 55) rather than precise measurements (e.g., 42.3, 53.7). This rounding introduces label noise, potentially limiting the model's ability to match targets accurately during validation.

Reagents

The codon-optimized genes encoding β-glucosidase from *Pyrococcus furiosus* (UniProt ID: Q51723) and *Thermoanaerobacterium aotearoense* (UniProt ID: A0A0H4NXH8) were synthesized and cloned into the pET-30a(+) vector by LifeTein, LLC (Somerset, NJ, USA), and used for the construction of mutant genes. *Escherichia coli* BL21(DE3) Electrocompetent Cells and p-nitrophenyl-β-D-glucopyranoside (pNP-Glc) were obtained from Sigma-Aldrich (St. Louis, MO, USA). Isopropyl-β-D-thiogalactopyranoside was purchased from Gold Biotechnology (St. Louis, MO, USA). All other chemicals were purchased from Fisher Scientific (Waltham, MA, USA), unless otherwise specified.

Expression and purification of *Pyrococcus furiosus* and *Thermoanaerobacterium aotearoense* β-glucosidase mutants

Several point mutants of *Pyrococcus furiosus* β-glucosidase (PfBGL) from our other unpublished studies were expressed and purified for k_{cat}/K_m measurements using pNP-Glc (**Table S1**). Similarly, *Thermoanaerobacterium aotearoense* (TaBGL) and its point mutants from our other unpublished studies were also expressed and purified (**Table S1**). *Escherichia coli* BL21(DE3) cells carrying the pET-30a(+) plasmids with *pfbgl* mutant genes, as well as the wild-type *tabgl* and its mutant genes, were cultured overnight in 10 mL of LB medium containing 50 μg/mL of kanamycin at 37 °C, with continuous shaking at 250 rpm in a New Brunswick Scientific Innova TM4230 incubator (Edison, NJ, USA). The starter culture was then transferred to 1 L of LB medium supplemented with 50 μg/mL of kanamycin and further incubated at the same temperature and shaking conditions. Once the optical density at 600 nm (OD600) reached approximately 0.6, protein expression was induced by adding isopropyl-β-D-thiogalactopyranoside to a final concentration of 1 mM. The culture was then incubated for ~16 hours at 25 °C with shaking at 250 rpm. Following the incubation, cells were harvested by centrifugation at 5,000 rpm for 20 minutes using a Beckman Coulter Avanti J-25 centrifuge (Brea, CA, USA), then stored at -80 °C until further processing. The cell pellets were resuspended in 50 mM phosphate buffer (pH 7.2, 50 mM Na_2HPO_4/NaH_2PO_4) and subjected to sonication in four 1-minute cycles with 1-minute intervals of rest, using a Hielscher UP200S ultrasonicator (Teltow, Germany). The lysates were then centrifuged at 14,000 rpm for one hour, and the supernatants were collected. The enzymes were purified from the supernatants by gravity-flow column chromatography, using HisLink Resin (Promega, Madison, WI, USA) and wash/elution buffers containing 25 mM and 200 mM imidazole, respectively. The eluted proteins were dialyzed twice against four liters of 50 mM phosphate buffer. After dialysis, the proteins were lyophilized overnight using a Labconco freeze-dry system (Kansas City, MO, USA) and stored at -80 °C. Protein purity was assessed via SDS-PAGE and determined to be >95 % for all samples. Protein concentrations were measured using the PierceTM BCA Protein Assay Kit (Thermo Fisher Scientific, Waltham, MA, USA).

Phylogenetic analysis and ancestral sequence reconstruction

Enzyme sequences were retrieved by a protein-protein basic local alignment search tool (BLASTp) search against the National Center for Biotechnology Information (NCBI) non-redundant database. The β-glucosidase sequence from *Pyrococcus furiosus* (UniProt ID: Q51723) was used as a query sequence for the search. Only homologs with >70% sequence identity were selected, and taxonomically redundant sequences were excluded. A total of 14 sequences were aligned using ClustalW and manually edited to adjust for misalignments, resulting in a multiple sequence alignment with nine protein sequences. The alignment was tested for the best model of protein evolution using MEGA 11.[9] The LG amino acid substitution model with a gamma distribution was used.[10] The phylogeny was constructed using a maximum likelihood tree.[11] The sequences of all ancestral nodes were deduced using the posterior probabilities of each amino acid being found at a specific residue. Then, SoluProt[12] and DeepSTABp[13] were utilized to confirm high solubility and melting temperatures of the ancestral sequences prior to their expression. The ancestral protein genes were codon-optimized, and synthetized and cloned into the pET-30a(+) vector by LifeTein, LLC. These ancestral proteins were expressed and purified as described above.

Enzyme assays

Initial reaction rates for the hydrolysis of pNP-Glc in citrate-phosphate buffer (100 mM citric acid/Na_2HPO_4, pH 6) were determined by measuring absorbance changes at 400 nm over time at designated temperatures as described elsewhere,[14, 15] using a Varian Cary 50 UV-VIS spectrophotometer (Palo Alto, CA, USA) equipped with a Quantum Northwest Peltier controlled temperature unit (Liberty Lake, WA, USA). During the measurements, the enzyme concentration was set at 20 nM. The molar extinction coefficient change ($\Delta\varepsilon$) for the substrate was measured to be 1,812 $M^{-1}cm^{-1}$ for pNP-Glc and directly used for the calculation of reaction rates, which were corrected for the spontaneous hydrolysis of pNP-Glc. The Michaelis-Menten kinetic parameters (k_{cat} and K_m) were determined from the Eadie-Hofstee plot.

Table S2. The characteristics of data used to train and validate ML models.

Enzyme	Number of Unique Protein Sequence Entries (Total Number of Unique Data Entries) [a]					Protein Sequence	
	Species	Biological Kingdom	Wild -type	Mutants	Total	Sequence Identity [b,c]	Number of Amino Acids [c]
β-glucosidase	96	archaea, animalia, bacteria, fungi, metagenome, plantae	119 (705)	141 (180)	260 (885)	0.34± 0.22	523 ±133

[a]The total number of unique data entries is greater than the number of unique protein sequence entries due to the presence of multiple k_{cat}/K_m values at different temperatures for a given protein sequence.
[b]The average sequence identity between a set of sequences was calculated by aligning each pair of sequences using the pairwise2 module from the Biopython library.
[c]Average ± one standard deviation

Table S3. The top 100 and bottom 100 ML model combinations within the three-module framework, ranked by R^2 for predicting protein sequence- and temperature-dependent k_{cat}/K_m values, calculated from the validation sets across ten rounds of random data splitting by protein sequence.

Rank	ML models[a]			R^{2} [,b]
	Module 1 (T_{opt})	Module 2 ($k_{cat}/K_{m,\,max}$)	Module 3 (relative k_{cat}/K_m vs. T profile	
1	ESM1b + XGB	ESM1b + SVR	BoW + RF	0.376937
2	ESM1b + XGB	ESM1b + SVR	B45 + RF	0.376395
3	BoW + RF	ESM1b + SVR	Four + ENR	0.376024
4	BoW + RF	ESM1b + SVR	Tri + ENR	0.376024
5	ESM1b + XGB	ESM1b + SVR	ESM1b + RF	0.375507
6	Tri + RF	ESM1b + SVR	Four + ENR	0.375505
7	Tri + RF	ESM1b + SVR	Tri + ENR	0.375505
8	B45 + XGB	ESM1b + SVR	Four + ENR	0.375456
9	B45 + XGB	ESM1b + SVR	Tri + ENR	0.375456
10	B62 + XGB	ESM1b + SVR	Four + ENR	0.375058
11	B62 + XGB	ESM1b + SVR	Tri + ENR	0.375058
12	OHE + XGB	ESM1b + SVR	Four + ENR	0.374699
13	OHE + XGB	ESM1b + SVR	Tri + ENR	0.374699
14	Bi + RF	ESM1b + SVR	Four + ENR	0.374554
15	Bi + RF	ESM1b + SVR	Tri + ENR	0.374554
16	OHE + XGB	ESM1b + SVR	Four + ENR	0.374468
17	OHE + XGB	ESM1b + SVR	Tri + ENR	0.374468
18	Bi + XGB	ESM1b + SVR	Four + ENR	0.374355
19	Bi + XGB	ESM1b + SVR	Tri + ENR	0.374355
20	Four + RF	ESM1b + SVR	Four + ENR	0.374168
21	Four + RF	ESM1b + SVR	Tri + ENR	0.374168
22	ESM1b + RF	ESM1b + SVR	Four + ENR	0.373874
23	ESM1b + RF	ESM1b + SVR	Tri + ENR	0.373874
24	Tri + LASSO	ESM1b + SVR	Four + ENR	0.373871
25	Tri + LASSO	ESM1b + SVR	Tri + ENR	0.373871
26	Four + LASSO	ESM1b + SVR	Four + ENR	0.373846
27	Four + LASSO	ESM1b + SVR	Tri + ENR	0.373846
28	ES1Mb + MLP	ESM1b + SVR	Four + ENR	0.373820
29	ES1Mb + MLP	ESM1b + SVR	Tri + ENR	0.373820
30	OHE + LASSO	ESM1b + SVR	BoW + RF	0.373819
31	ESM1b + XGB	ESM1b + SVR	Four + ENR	0.373757
32	ESM1b + XGB	ESM1b + SVR	Tri + ENR	0.373757
33	OHE + LASSO	OHE + XGB	BoW + RF	0.373607
34	ESM1b + XGB	OHE + XGB	B45 + RF	0.373570

35	OHE + LASSO	ESM1b + SVR	Four + ENR	0.373272
36	OHE + LASSO	ESM1b + SVR	Tri + ENR	0.373272
37	ESM1b + XGB	OHE + XGB	ESM1b + RF	0.373149
38	Four + XGB	ESM1b + SVR	Four + ENR	0.373088
39	Four + XGB	ESM1b + SVR	Tri + ENR	0.373088
40	Bi + LASSO	ESM1b + SVR	Four + ENR	0.373013
41	Bi + LASSO	ESM1b + SVR	Tri + ENR	0.373013
42	ESM1b + LASSO	ESM1b + SVR	BoW + RF	0.372999
43	B45 + RF	ESM1b + SVR	Four + ENR	0.372985
44	B45 + RF	ESM1b + SVR	Tri + ENR	0.372985
45	B62 + RF	ESM1b + SVR	Four + ENR	0.372860
46	B62 + RF	ESM1b + SVR	Tri + ENR	0.372860
47	ESM1b + XGB	ESM1b + SVR	B62 + RF	0.372800
48	ESM1b + RF	ESM1b + SVR	BoW + RF	0.372655
49	ESM1b + LASSO	ESM1b + SVR	Four + ENR	0.372371
50	ESM1b + LASSO	ESM1b + SVR	Tri + ENR	0.372371
51	OHE + LASSO	ESM1b + SVR	ESM1b + RF	0.371877
52	OHE + LASSO	OHE + XGB	B45 + RF	0.371850
53	OHE + LASSO	OHE + XGB	ESM1b + RF	0.371834
54	ESM1b + XGB	OHE + XGB	BoW + RF	0.371602
55	ESM1b + RF	ESM1b + SVR	ESM1b + RF	0.371589
56	B62 + RF	ESM1b + SVR	BoW + RF	0.371530
57	OHE + LASSO	OHE + XGB	Tri + RF	0.370676
58	ESM1b + RF	ESM1b + SVR	B45 + RF	0.370230
59	OHE + XGB	ESM1b + SVR	BoW + RF	0.370006
60	B45 + RF	ESM1b + SVR	BoW + RF	0.369989
61	B45 + XGB	ESM1b + SVR	BoW + RF	0.369833
62	OHE + LASSO	ESM1b + SVR	B45 + RF	0.369811
63	ESM1b + XGB	ESM1b + SVR	OHE + XGB	0.369714
64	ESM1b + LASSO	ESM1b + SVR	ESM1b + RF	0.369671
65	OHE + XGB	OHE + XGB	BoW + RF	0.369153
66	ESM1b + XGB	ESM1b + SVR	Tri + RF	0.368732
67	ESM1b + LASSO	ESM1b + SVR	B45 + RF	0.368478
68	BoW + RF	OHE + XGB	Four + ENR	0.368405
69	BoW + RF	OHE + XGB	Tri + ENR	0.368405
70	ESM1b + XGB	OHE + XGB	B62 + RF	0.368359
71	ESM1b + LASSO	OHE + XGB	BoW + RF	0.368232
72	OHE + LASSO	ESM1b + SVR	Tri + RF	0.368102
73	BoW + RF	ESM1b + RF	Four + ENR	0.368064
74	BoW + RF	ESM1b + RF	Tri + ENR	0.368064

75	ESM1b + RF	ESM1b + SVR	B62 + RF	0.368002
76	ESM1b + XGB	ESM1b + RF	ESM1b + RF	0.367745
77	B45 + XGB	OHE + XGB	Four + ENR	0.367635
78	B45 + XGB	OHE + XGB	Tri + ENR	0.367635
79	ESM1b + RF	ESM1b + SVR	Tri + RF	0.367633
80	Bi + LASSO	ESM1b + SVR	BoW + RF	0.367462
81	B45 + XGB	ESM1b + RF	Four + ENR	0.367224
82	B45 + XGB	ESM1b + RF	Tri + ENR	0.367224
83	ES1Mb + MLP	OHE + XGB	Four + ENR	0.367220
84	ES1Mb + MLP	OHE + XGB	Tri + ENR	0.367220
85	Bi + LASSO	OHE + XGB	BoW + RF	0.367191
86	B45 + XGB	ESM1b + SVR	B45 + RF	0.367136
87	OHE + LASSO	OHE + XGB	B62 + RF	0.366790
88	ESM1b + RF	OHE + XGB	Four + ENR	0.366775
89	ESM1b + RF	OHE + XGB	Tri + ENR	0.366775
90	Tri + RF	ESM1b + RF	Four + ENR	0.366739
91	Tri + RF	ESM1b + RF	Tri + ENR	0.366739
92	B45 + XGB	ESM1b + SVR	ESM1b + RF	0.366611
93	ESM1b + RF	OHE + XGB	ESM1b + RF	0.366485
94	ESM1b + XGB	OHE + XGB	OHE + XGB	0.366423
95	ESM1b + XGB	OHE + XGB	Four + ENR	0.366405
96	ESM1b + XGB	OHE + XGB	Tri + ENR	0.366405
97	ESM1b + LASSO	OHE + XGB	ESM1b + RF	0.366361
98	OHE + XGB	ESM1b + SVR	ESM1b + RF	0.366303
99	B62 + XGB	ESM1b + RF	Four + ENR	0.366245
100	B62 + XGB	ESM1b + RF	Tri + ENR	0.366245
7901	Tri + RF	BoW + MLP	Four + XGB	0.153582
7902	Bi + XGB	Four + XGB	Tri + XGB	0.153433
7903	B45 + XGB	Four + XGB	Four + XGB	0.153387
7904	Four + XGB	BoW + RF	Tri + XGB	0.153371
7905	OHE + LASSO	Four + XGB	Bi + XGB	0.153354
7906	BoW + RF	BoW + MLP	B45 + XGB	0.153306
7907	OHE + XGB	Four + XGB	Tri + XGB	0.153045
7908	Tri + RF	Four + XGB	Four + RF	0.152752
7909	OHE + XGB	Four + XGB	Four + XGB	0.151790
7910	Tri + RF	BoW + MLP	B62 + XGB	0.151634
7911	Bi + XGB	Four + XGB	Four + XGB	0.151381
7912	Four + LASSO	Four + XGB	BoW + XGB	0.151050
7913	Bi + RF	Four + XGB	Tri + XGB	0.150567
7914	Tri + RF	BoW + MLP	B45 + XGB	0.150525

7915	Four + XGB	Four + XGB	BoW + XGB	0.150489
7916	Tri + RF	BoW + RF	B45 + XGB	0.150476
7917	Four + XGB	BoW + RF	B45 + XGB	0.150367
7918	OHE + XGB	Four + XGB	B45 + XGB	0.150052
7919	B62 + RF	Four + XGB	Bi + XGB	0.149823
7920	Tri + RF	BoW + MLP	Tri + XGB	0.149790
7921	Tri + RF	Four + XGB	BoW + XGB	0.149252
7922	Bi + RF	Four + XGB	OHE + XGB	0.149073
7923	Four + XGB	BoW + MLP	B45 + XGB	0.148769
7924	ESM1b + LASSO	Four + XGB	B62 + XGB	0.148744
7925	OHE + XGB	Four + XGB	B62 + XGB	0.148467
7926	B45 + XGB	Four + XGB	Bi + XGB	0.148422
7927	B45 + RF	Four + XGB	Bi + XGB	0.148409
7928	Bi + RF	Four + XGB	Four + XGB	0.147905
7929	ESM1b + LASSO	Four + XGB	B45 + XGB	0.147890
7930	ESM1b + LASSO	Four + XGB	Four + XGB	0.147870
7931	BoW + RF	Four + XGB	Four + RF	0.147721
7932	OHE + LASSO	Four + XGB	B62 + XGB	0.147443
7933	Tri + LASSO	BoW + MLP	B45 + XGB	0.146959
7934	BoW + RF	Four + XGB	BoW + XGB	0.146627
7935	Tri + LASSO	BoW + RF	B45 + XGB	0.146305
7936	ESM1b + XGB	Four + XGB	Bi + XGB	0.146114
7937	Four + RF	Four + XGB	BoW + XGB	0.145555
7938	Four + XGB	BoW + MLP	B62 + XGB	0.145545
7939	ES1Mb + MLP	Four + XGB	B62 + XGB	0.144455
7940	Bi + XGB	Four + XGB	B62 + XGB	0.144285
7941	Four + LASSO	Four + XGB	OHE + XGB	0.143828
7942	ES1Mb + MLP	Four + XGB	OHE + XGB	0.143345
7943	B45 + XGB	Four + XGB	B62 + XGB	0.143222
7944	Bi + XGB	Four + XGB	Bi + XGB	0.142716
7945	Tri + LASSO	Four + XGB	OHE + XGB	0.142591
7946	B62 + XGB	Four + XGB	B45 + XGB	0.142445
7947	Bi + LASSO	Four + XGB	B62 + XGB	0.142371
7948	ES1Mb + MLP	Four + XGB	Four + XGB	0.141098
7949	Four + RF	Four + XGB	OHE + XGB	0.140906
7950	OHE + XGB	Four + XGB	Bi + XGB	0.140900
7951	Four + RF	Four + XGB	Four + XGB	0.140235
7952	B62 + XGB	Four + XGB	OHE + XGB	0.140124
7953	Four + XGB	BoW + MLP	Tri + XGB	0.140053
7954	ES1Mb + MLP	Four + XGB	B45 + XGB	0.140034

7955	Tri + RF	Four + XGB	OHE + XGB	0.139798
7956	Four + LASSO	Four + XGB	Four + XGB	0.139135
7957	ESM1b + RF	Four + XGB	Bi + XGB	0.138851
7958	Bi + RF	Four + XGB	B62 + XGB	0.137803
7959	B62 + XGB	Four + XGB	Bi + XGB	0.137641
7960	Bi + LASSO	Four + XGB	Bi + XGB	0.137260
7961	B62 + XGB	Four + XGB	Four + XGB	0.136991
7962	Four + RF	Four + XGB	Tri + XGB	0.136952
7963	Four + XGB	Four + XGB	Four + XGB	0.136908
7964	B62 + XGB	Four + XGB	B62 + XGB	0.135770
7965	Four + XGB	Four + XGB	Bi + XGB	0.133822
7966	OHE + LASSO	Four + XGB	B45 + XGB	0.133726
7967	Four + LASSO	Four + XGB	Bi + XGB	0.133103
7968	Tri + LASSO	Four + XGB	Tri + XGB	0.133081
7969	Four + XGB	Four + XGB	OHE + XGB	0.132959
7970	Bi + RF	Four + XGB	B45 + XGB	0.132273
7971	Tri + RF	Four + XGB	Four + XGB	0.131810
7972	Four + LASSO	Four + XGB	B62 + XGB	0.131030
7973	BoW + RF	Four + XGB	Tri + XGB	0.130978
7974	B62 + XGB	Four + XGB	Tri + XGB	0.130346
7975	Four + RF	Four + XGB	B62 + XGB	0.130151
7976	Four + LASSO	Four + XGB	Tri + XGB	0.130134
7977	Tri + RF	Four + XGB	Tri + XGB	0.129410
7978	Tri + LASSO	Four + XGB	Four + XGB	0.129325
7979	Bi + XGB	Four + XGB	B45 + XGB	0.129278
7980	BoW + RF	Four + XGB	Four + XGB	0.128676
7981	ESM1b + LASSO	Four + XGB	Bi + XGB	0.128493
7982	Bi + RF	Four + XGB	Bi + XGB	0.128252
7983	Tri + RF	Four + XGB	Bi + XGB	0.127985
7984	Four + RF	Four + XGB	B45 + XGB	0.127782
7985	Four + XGB	Four + XGB	Tri + XGB	0.127689
7986	Bi + LASSO	Four + XGB	B45 + XGB	0.126667
7987	Tri + RF	Four + XGB	B62 + XGB	0.125497
7988	Four + RF	Four + XGB	Bi + XGB	0.125179
7989	Tri + LASSO	Four + XGB	Bi + XGB	0.124932
7990	BoW + RF	Four + XGB	B62 + XGB	0.124501
7991	Tri + LASSO	Four + XGB	B62 + XGB	0.123827
7992	Four + XGB	Four + XGB	B45 + XGB	0.121823
7993	Tri + RF	Four + XGB	B45 + XGB	0.120252
7994	ES1Mb + MLP	Four + XGB	Bi + XGB	0.118729

7995	Four + XGB	Four + XGB	B62 + XGB	0.117579
7996	Four + LASSO	Four + XGB	B45 + XGB	0.116567
7997	BoW + RF	Four + XGB	OHE + XGB	0.113817
7998	Tri + LASSO	Four + XGB	B45 + XGB	0.109598
7999	BoW + RF	Four + XGB	B45 + XGB	0.108848
8000	BoW + RF	Four + XGB	Bi + XGB	0.106484

[a]The ML models in the three modules were trained individually, which were then integrated under the three-module framework. The R^2 values were determined using the validation dataset, where k_{cat}/K_m values were calculated from the ML models in Module 3, with T_{opt} and $k_{cat}/K_{m,\ max}$ values predicted from the ML models in Modules 1 and 2, respectively.
[b]The R^2 values represent the averages calculated from the validation sets across ten rounds of random data splitting by protein sequence.

Abbreviation for protein sequence representations: BoW: Bag-of-Words; B45: BLOSUM45; Four: Fourgram; Tri: Trigram; B62: BLOSUM62; Bi: Bigram; OHE: One-Hot-Encoder

Abbreviation for regression algorithms: XGB: Extreme Gradient Boosting; SVR: Support Vector Regression; RF: Random Forest; ENR: Elastic Net Regression; LASSO: Least Absolute Shrinkage and Selection Operator; MLP: Multi-Layer Perceptron

Table S4. Comparison between the three- and single-module ML frameworks for protein sequence- and temperature-dependent k_{cat}/K_m prediction in terms of various evaluation metrics calculated using validation and training datasets. The evaluation metric values shown here represent the average values from ten rounds of random data splitting by protein sequence (**Table S5**). XGB: Extreme Gradient Boosting; SVR: Support Vector Regression; BoW: Bag-of-Words; RF: Random Forest; B45: BLOSUM45; B62: BLOSUM62; Bi: Bigram.

**Validation dataset**				R^2	**PCC**	**MAE**	**RMSE**
	**1st Module**	**2nd Module**	**3rd Module**				
**Three module framework**[a]	ESM1b + XGB	ESM1b + SVR	BoW + RF	0.376937	0.634688	0.74952	1.018436
	ESM1b + XGB	ESM1b + SVR	B45 + RF	0.376395	0.633895	0.747968	1.018849
**Single module framework**	B62 + RF			0.393984	0.655816	0.699171	0.993301
	ESM1b + SVR			0.378481	0.637037	0.737383	1.012977
	Bi + SVR			0.354715	0.62799	0.759231	1.034182

[a]The ML models in the three modules were trained individually, which were then integrated under the three-module framework. The R^2, PCC, MAE, and RMSE values were determined using the validation dataset, where k_{cat}/K_m values were calculated from the ML models in Module 3, with T_{opt} and $k_{cat}/K_{m,\ max}$ values predicted from the ML models in Modules 1 and 2, respectively.

**Training dataset**				R^2	**PCC**	**MAE**	**RMSE**
	**1st Module**	**2nd Module**	**3rd Module**				
**Three module ML framework**[a]	ESM1b + XGB	ESM1b + SVR	BoW + RF	0.618594	0.79951	0.567781	0.806266
	ESM1b + XGB	ESM1b + SVR	B45 + RF	0.614427	0.796787	0.569302	0.810672
**Single module ML framework**	B62 + RF			0.983159	0.991865	0.092762	0.169295
	ESM1b + SVR			0.828101	0.916145	0.361889	0.541086
	Bi + SVR			0.880022	0.941885	0.296282	0.452249

[a]The ML models in the three modules were trained individually, which were then integrated under the three-module framework. The R^2, PCC, MAE, and RMSE values were determined using the training dataset, where k_{cat}/K_m values were calculated from the ML models in Module 3, with T_{opt} and $k_{cat}/K_{m,\ max}$ values predicted from the ML models in Modules 1 and 2, respectively.

Table S5. The number of data entries in the training and validation sets across ten rounds of random data splitting based on protein sequences.

	Round									
	**1**	**2**	**3**	**4**	**5**	**6**	**7**	**8**	**9**	**10**
**Training**	743	731	722	722	733	721	758	716	709	687
**Validation**	142	154	163	163	152	164	127	169	176	198

Table S6. Comparison between the three- and single-module ML frameworks for protein sequence- and temperature-dependent k_{cat}/K_m prediction in terms of various evaluation metrics calculated using the subsets of validation sets. The evaluation metric values shown here represent the averages calculated from the validation sets across ten rounds of random data splitting by protein sequence (**Table S7**). XGB: Extreme Gradient Boosting; SVR: Support Vector Regression; BoW: Bag-of-Words; RF: Random Forest; B45: BLOSUM45; B62: BLOSUM62; Bi: Bigram.

Validation dataset				R^2				
				Total	Wild-type vs. mutant		Maximum sequence identity	
	1st Module	2nd Module	3rd Module		Wild-type	Mutant	≥ 80 %	< 80%
Three module ML framework [a]	ESM1b + XGB	ESM1b + SVR	BoW + RF	0.376937	0.168406	0.681149	0.584264	0.190456
	ESM1b + XGB	ESM1b + SVR	B45 + RF	0.376395	0.167919	0.681936	0.583365	0.194858
Single module ML framework	B62 + RF			0.393984	0.157146	0.770851	0.753481	0.061942
	ESM1b + SVR			0.378481	0.176996	0.677202	0.674627	0.111431
	Bi + SVR			0.354715	0.13725	0.687825	0.682152	0.036366

[a]The ML models in the three modules were trained individually, which were then integrated under the three-module framework. The R^2 values were determined using the validation dataset, where k_{cat}/K_m values were calculated from the ML models in Module 3, with T_{opt} and $k_{cat}/K_{m,\ max}$ values predicted from the ML models in Modules 1 and 2, respectively.

Validation dataset				PCC				
				Total	Wild-type vs. mutant		Maximum sequence identity	
	1st Module	2nd Module	3rd Module		Wild-type	Mutant	≥ 80 %	< 80%
Three module ML framework [a]	ESM1b + XGB	ESM1b + SVR	BoW + RF	0.634688	0.483369	0.849198	0.795677	0.499609
	ESM1b + XGB	ESM1b + SVR	B45 + RF	0.633895	0.480142	0.85093	0.794239	0.500933
Single module ML framework	B62 + RF			0.655816	0.477523	0.890027	0.881928	0.409943
	ESM1b + SVR			0.637037	0.47601	0.859759	0.841977	0.434611
	Bi + SVR			0.62799	0.443021	0.860096	0.846021	0.411386

[a]The ML models in the three modules were trained individually, which were then integrated under the three-module framework. The PCC values were determined using the validation dataset, where k_{cat}/K_m values were calculated from the ML models in Module 3, with T_{opt} and $k_{cat}/K_{m,\ max}$ values predicted from the ML models in Modules 1 and 2, respectively.

Validation dataset				MAE				
				Total	Wild-type vs. mutant		Maximum sequence identity	
Three module ML framework[a]	1st Module	2nd Module	3rd Module		Wild-type	Mutant	≥ 80 %	< 80%
	ESM1b + XGB	ESM1b + SVR	BoW + RF	0.74952	0.807804	0.558016	0.684699	0.778454
	ESM1b + XGB	ESM1b + SVR	B45 + RF	0.747968	0.807495	0.552537	0.681765	0.776298
Single module ML framework	B62 + RF			0.761334	0.878387	0.358966	0.395351	0.8903
	ESM1b + SVR			0.701666	0.770312	0.465695	0.479829	0.779837
	Bi + SVR			0.758755	0.849601	0.446473	0.464265	0.862528

[a]The ML models in the three modules were trained individually, which were then integrated under the three-module framework. The MAE values were determined using the validation dataset, where k_{cat}/K_m values were calculated from the ML models in Module 3, with T_{opt} and $k_{cat}/K_{m, max}$ values predicted from the ML models in Modules 1 and 2, respectively.

Validation dataset				RMSE				
				Total	Wild-type vs. mutant		Maximum sequence identity	
Three module ML framework[a]	1st Module	2nd Module	3rd Module		Wild-type	Mutant	≥ 80 %	< 80%
	ESM1b + XGB	ESM1b + SVR	BoW + RF	1.018436	1.072334	0.787892	0.932017	1.02681
	ESM1b + XGB	ESM1b + SVR	B45 + RF	1.018849	1.072834	0.786656	0.933287	1.023644
Single module ML framework	B62 + RF			0.993301	1.066741	0.65914	0.692584	1.097134
	ESM1b + SVR			1.012977	1.06333	0.792114	0.820645	1.079071
	Bi + SVR			1.034182	1.092754	0.779967	0.809737	1.119366

[a]The ML models in the three modules were trained individually, which were then integrated under the three-module framework. The RMSE values were determined using the validation dataset, where k_{cat}/K_m values were calculated from the ML models in Module 3, with T_{opt} and $k_{cat}/K_{m, max}$ values predicted from the ML models in Modules 1 and 2, respectively.

Table S7. The number of data entries in subsets of the validation sets across ten rounds of random data splitting based on protein sequences.

	Round									
	**1**	**2**	**3**	**4**	**5**	**6**	**7**	**8**	**9**	**10**
**Wild-type**	110	112	127	121	115	123	97	132	138	158
**Mutant**	32	42	36	42	37	41	30	37	38	40
**>80 % maximum sequence identity**	37	33	63	67	65	54	41	60	50	88
**< 80% maximum sequence identity**	105	121	100	96	87	110	86	109	126	110
**Total**	142	154	163	163	152	164	127	169	176	198

Table S8. The pairwise sequence identity of Nodes 13 and 15 with *Pyrococcus furiosus* β-glucosidase (PfBGL) and *Thermococcus pacificus* β-glucosidase (TpBGL).

	PfBGL	TpBGL
Node 13	82.92	86.80
Node 15	87.01	83.09

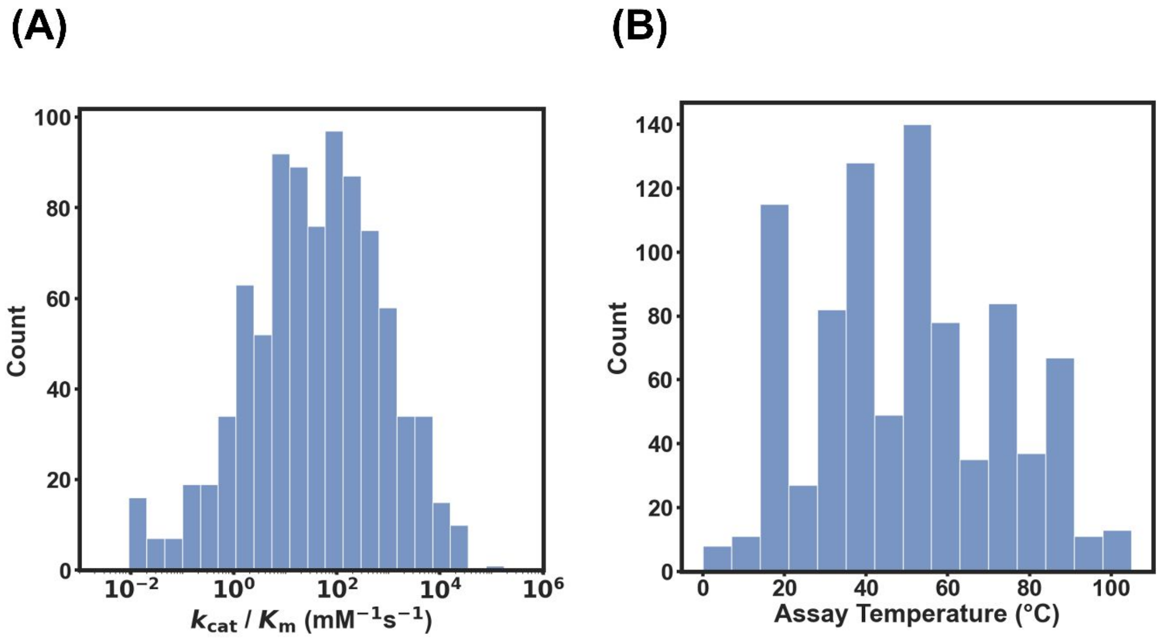

Figure S1. Characteristics of β-glucosidase data distribution in terms of (A) enzyme activity (k_{cat}/K_m) for the hydrolysis of pNP-Glc and (B) temperature at which k_{cat}/K_m was measured.

Entire dataset

Protein Sequence	k_{cat}/K_m ($mM^{-1}s^{-1}$) at the following temperatures (°C)							
	20	30	40	50	60	70	80	90
…KDEVEEIRKKD…	20	38	62	91	72	56	39	
…EDEVRAIRE…		120		520		1,220		735
…ADRAAAEAA…	2.1	3.6	1.5	0.2				
…LKGRVDW…		850			5,300			320
…RDDLQGKVDW…	0.05	1.6	1.3					

T_{opt} dataset

Protein Sequence	T_{opt}
…KDEVEEIRKKD…	50
…EDEVRAIRE…	70
…ADRAAAEAA…	30
…LKGRVDW…	60
…RDDLQGKVDW…	30

$k_{cat}/K_{m,max}$ dataset

Protein Sequence	$k_{cat}/K_{m,max}$
…KDEVEEIRKKD…	91
…EDEVRAIRE…	1,220
…ADRAAAEAA…	3.6
…LKGRVDW…	5,300
…RDDLQGKVDW…	1.6

k_{cat}/K_m vs. T profile dataset

Protein Sequence	Normalized k_{cat}/K_m at the following $T - T_{opt}$							
	-40	-30	-20	-10	0	10	20	30
…KDEVEEIRKKD…		0.22	0.42	0.68	1	0.79	0.62	0.43
…EDEVRAIRE…	0.10		0.43		1		0.60	
…ADRAAAEAA…				0.58	1	0.42	0.06	
…LKGRVDW…		0.16			1			0.06
…RDDLQGKVDW…				0.03	1	0.81		

Training set   Validation set

Figure S2. Example of sequence-based data splitting for training and validation. Multiple k_{cat}/K_m values exist at different temperatures for many BGL sequences. For each collected BGL sequence, all k_{cat}/K_m data were used exclusively for either training or validation, but not both. Consequently, the BGL sequences in the validation set remained unseen during training.

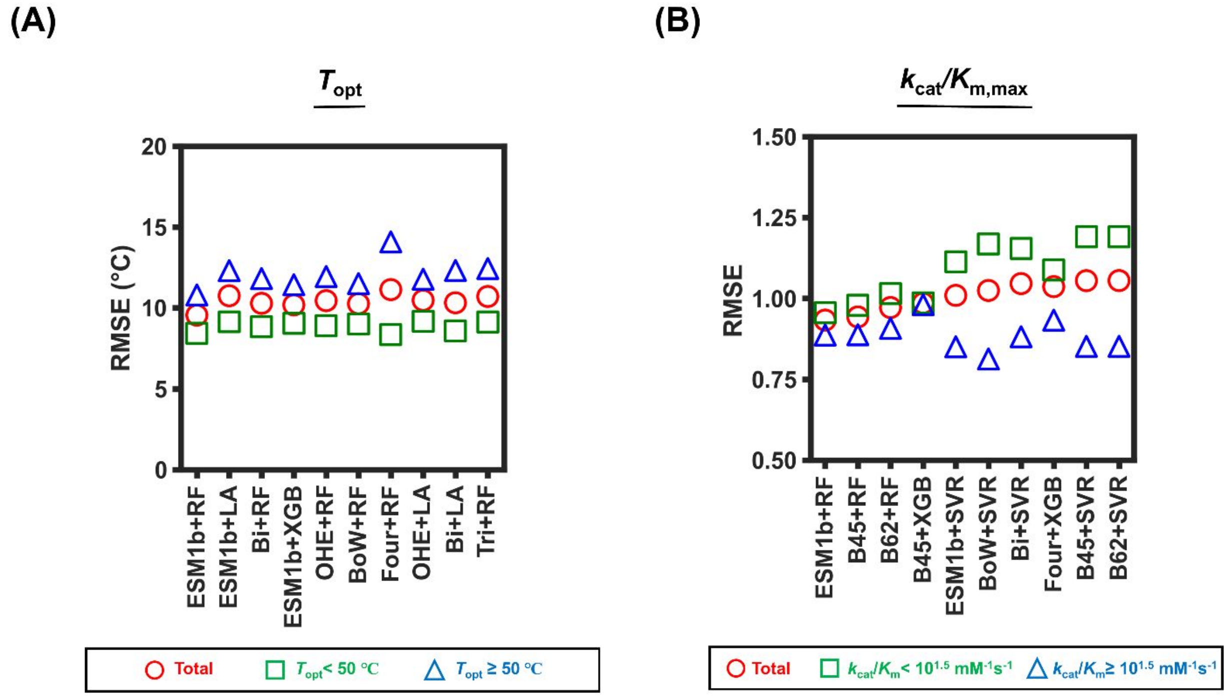

Figure S3. Prediction performance of the top ML models in Modules 1 and 2 on the validation subsets, grouped based on (A) T_{opt} and (B) $k_{cat}/K_{m,\ max}$ values. The RMSE value is presented as an evaluation metric, representing the average calculated from the validation sets across ten rounds of random data splitting by protein sequence. The validation set was divided into two subsets based on: (A) T_{opt} = 50 °C and (B) $k_{cat}/K_{m,max} = 10^{1.5}$ $mM^{-1}s^{-1}$. These cutoff values were chosen to ensure approximately equal numbers of data entries in each subset. We did not perform a similar analysis with more than two subgroups in each case due to the limited number of data entries per subgroup. The RMSE value shown in (B) is for log ($k_{cat}/K_{m,max}$) and is therefore unitless. The sequence representation methods shown here include ESM1b, Bigram (Bi), One-Hot-Encoder (OHE), Bag-of-Words (BoW), Fourgram (Four), Trigram (Tri), BLOSUM45 (B45), and BLOSUM62 (B62). The regression algorithms shown here include Random Forest (RF), LASSO (LA), Extreme Gradient Boosting (XGB), and Support Vector Regression (SVR).

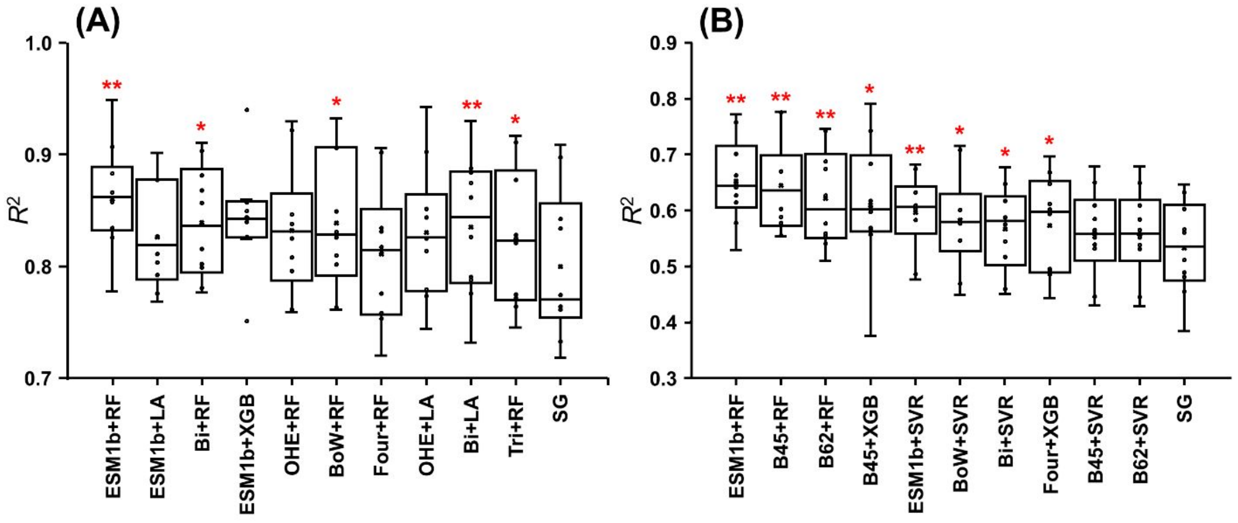

Figure S4. Comparison of the top ML models with sequence similarity-based geometric averaging calculations for (A) Module 1 (T_{opt}) and (B) Module 2 ($k_{cat}/K_{m,\ max}$) over ten rounds of random sequence-based data splitting. For this analysis, the entire dataset was randomly divided into five subsets by sequence, with 80% used for training and the remaining 20% for validation in each round of random splitting. This data splitting was independent of that used in the five-fold cross-validation (**Figure 2**), while still ensuring that no identical amino acid sequences were present in both the training and validation sets. The R^2 values shown here represent the averages calculated from the validation sets across ten rounds of random data splitting by protein sequence, with error bars indicating one standard deviation. The R^2 values of the top ML models for Modules 1 and 2 on the validation sets generated from this random data splitting were comparable to those obtained from the five-fold cross-validation (**Figure 2A-B**), confirming that the performance of the top models for unseen sequences was not strongly dependent on the selection of datasets for training and validation. *: $p<0.05$ and **: $p<0.01$ from a two-sided Wilcoxon signed-rank test, compared to the sequence similarity-based geometric averaging calculations (SG). The sequence representation methods shown here include ESM1b, Bigram (Bi), One-Hot-Encoder (OHE), Bag-of-Words (BoW), Fourgram (Four), Trigram (Tri), BLOSUM45 (B45), and BLOSUM62 (B62). The regression algorithms shown here include Random Forest (RF), LASSO (LA), Extreme Gradient Boosting (XGB), and Support Vector Regression (SVR).

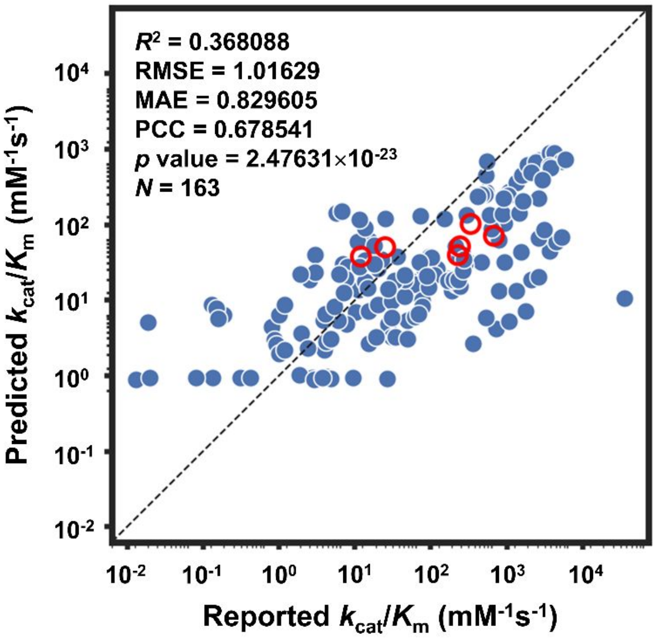

Figure S5. Correlation between predicted and reported k_{cat}/K_m values across various protein sequences and temperatures, when ESM1b+XGB for Module 1, ESM1b+SVR for Module 2, and B45+RF for Module 3 were utilized. For this illustration, the most representative validation dataset from the ten rounds of data splitting was selected, based on its R^2 value being closest to the average R^2 value calculated from the validation sets across the ten rounds. The RMSE, MAE, PCC, and p values were calculated from the most representative validation dataset. The red circles represent data for the two ancestral sequences. XGB: Extreme Gradient Boosting; SVR: Support Vector Regression; B45: BLOSUM45; RF: Random Forest.

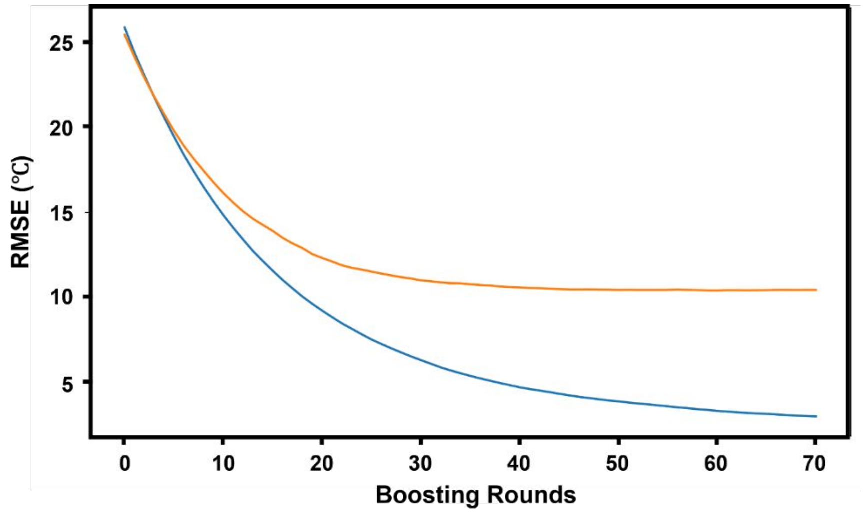

Figure S6. Validation loss curves of the hyperparameter-tuned XGBoost regressor using the ESM-1b sequence representation method for predicting the optimum temperature (*T_{opt}*) of β-glucosidase. The plot shows the Root Mean Square Error (RMSE) across boosting rounds for both training (blue) and validation (orange) sets. The model was trained using the following tuned parameters: L2 regularization (lambda) = 6.05, L1 regularization (alpha) = 61.6, minimum loss reduction for further partitioning (gamma) = 18.8, maximum tree depth (max_depth) = 8, minimum child weight = 25, subsample ratio = 0.829, column subsample ratio (colsample_bytree) = 0.6, and learning rate (eta) = 0.0887.

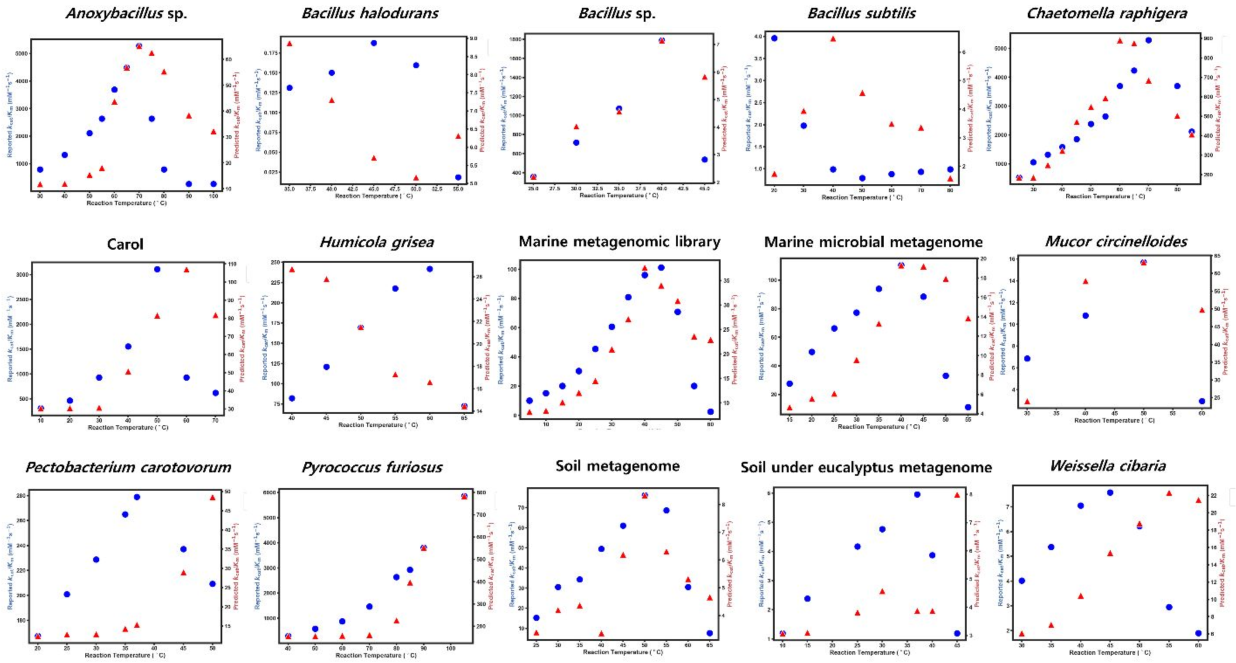

Figure S7. Comparison of predicted (red) and reported (blue) k_{cat}/K_m values as a function of protein sequence and temperature for the three-module framework consisting of ESM1b+XGB for Module 1, ESM1b+SVR for Module 2, and BoW+RF for Module 3. For the illustrations shown here, the most representative validation dataset from the ten rounds of data splitting was selected, based on its R^2 value being closest to the average R^2 value calculated from the validation sets across the ten rounds. XGB: Extreme Gradient Boosting; SVR: Support Vector Regression; BoW: Bag-of-Words; RF: Random Forest.

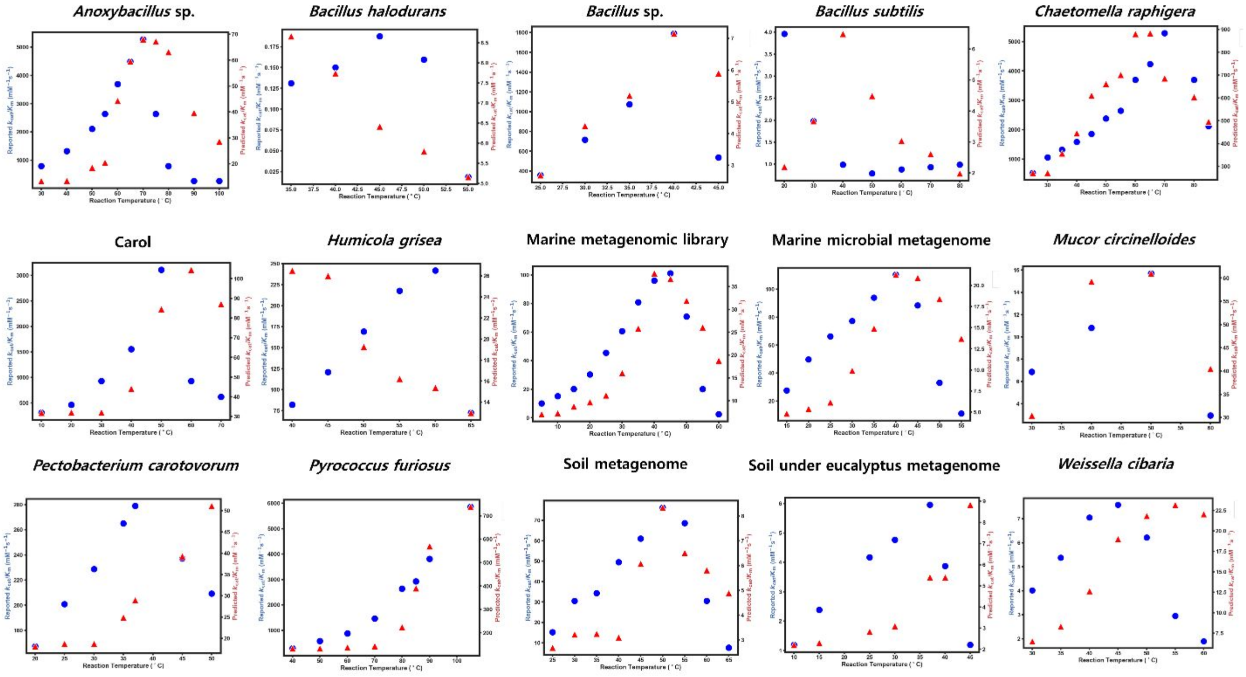

Figure S8. Comparison of predicted (red) and reported (blue) k_{cat}/K_m values as a function of protein sequence and temperature for the three-module framework consisting of ESM1b+XGB for Module 1, ESM1b+SVR for Module 2, and B45+RF for Module 3. For the illustrations shown here, the most representative validation dataset from the ten rounds of data splitting was selected, based on its R^2 value being closest to the average R^2 value calculated from the validation sets across the ten rounds. XGB: Extreme Gradient Boosting; SVR: Support Vector Regression; B45: BLOSUM45; RF: Random Forest.

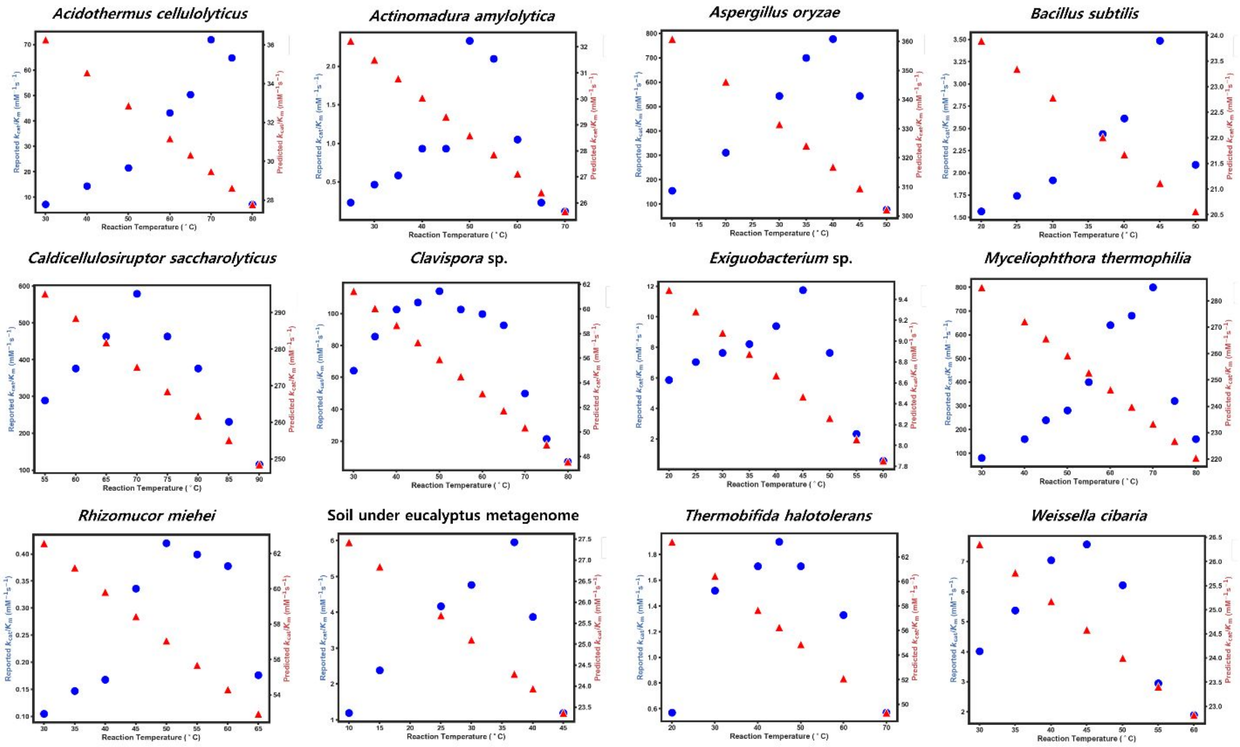

Figure S9. Comparison of predicted (red) and reported (blue) k_{cat}/K_m values as a function of protein sequence and temperature for the three-module framework consisting of BoW+RF for Module 1, ESM1b+SVR for Module 2, and Four+ENR for Module 3. For the illustrations shown here, the most representative validation dataset from the ten rounds of data splitting was selected, based on its R^2 value being closest to the average R^2 value calculated from the validation sets across the ten rounds. BoW: Bag-of-Words; RF: Random Forest; SVR: Support Vector Regression; Four: Fourgram; ENR: Elastic Net Regression.

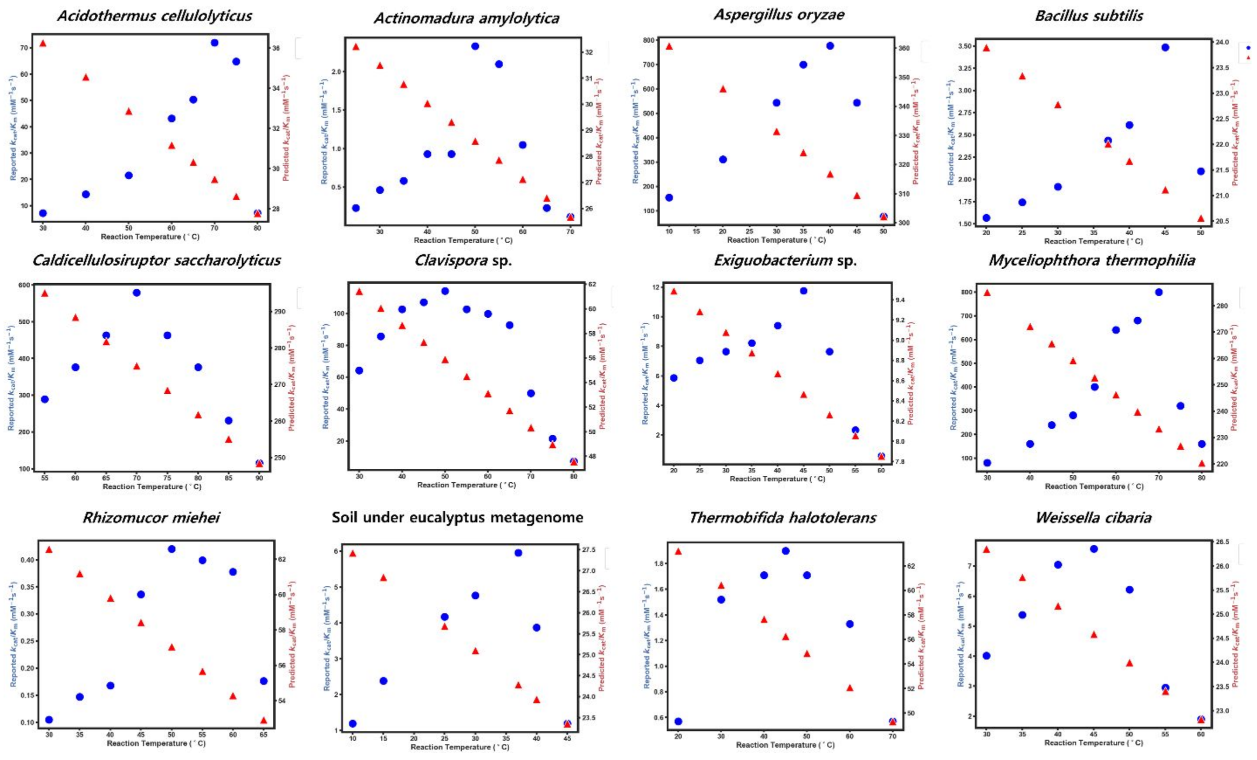

Figure S10. Comparison of predicted (red) and reported (blue) k_{cat}/K_m values as a function of protein sequence and temperature for the three-module framework consisting of BoW+RF for Module 1, ESM1b+SVR for Module 2, and Tri+ENR for Module 3. For the illustrations shown here, the most representative validation dataset from the ten rounds of data splitting was selected, based on its R^2 value being closest to the average R^2 value calculated from the validation sets across the ten rounds. BoW: Bag-of-Words; RF: Random Forest; SVR: Support Vector Regression; Tri: Trigram; ENR: Elastic Net Regression.

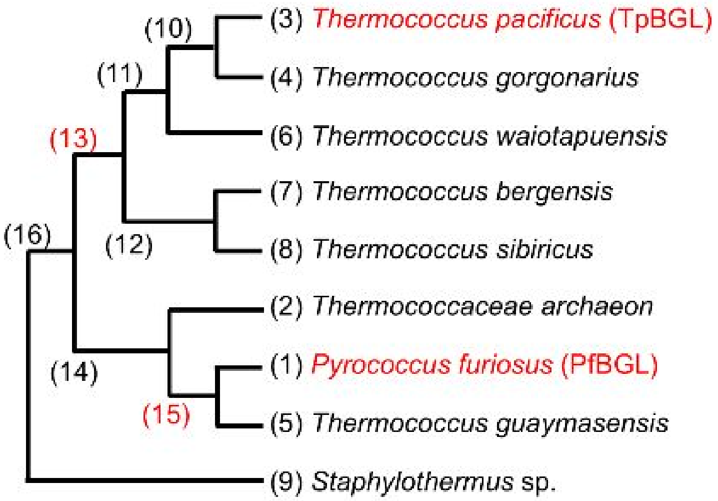

Figure S11. The phylogenetic analysis involving PfBGL. For this analysis, BGL sequences with a pairwise sequence identity (SI) of > 70% relative to PfBGL were used. PfBGL, TpBGL, Node 13, and Node 15 are shown in red.

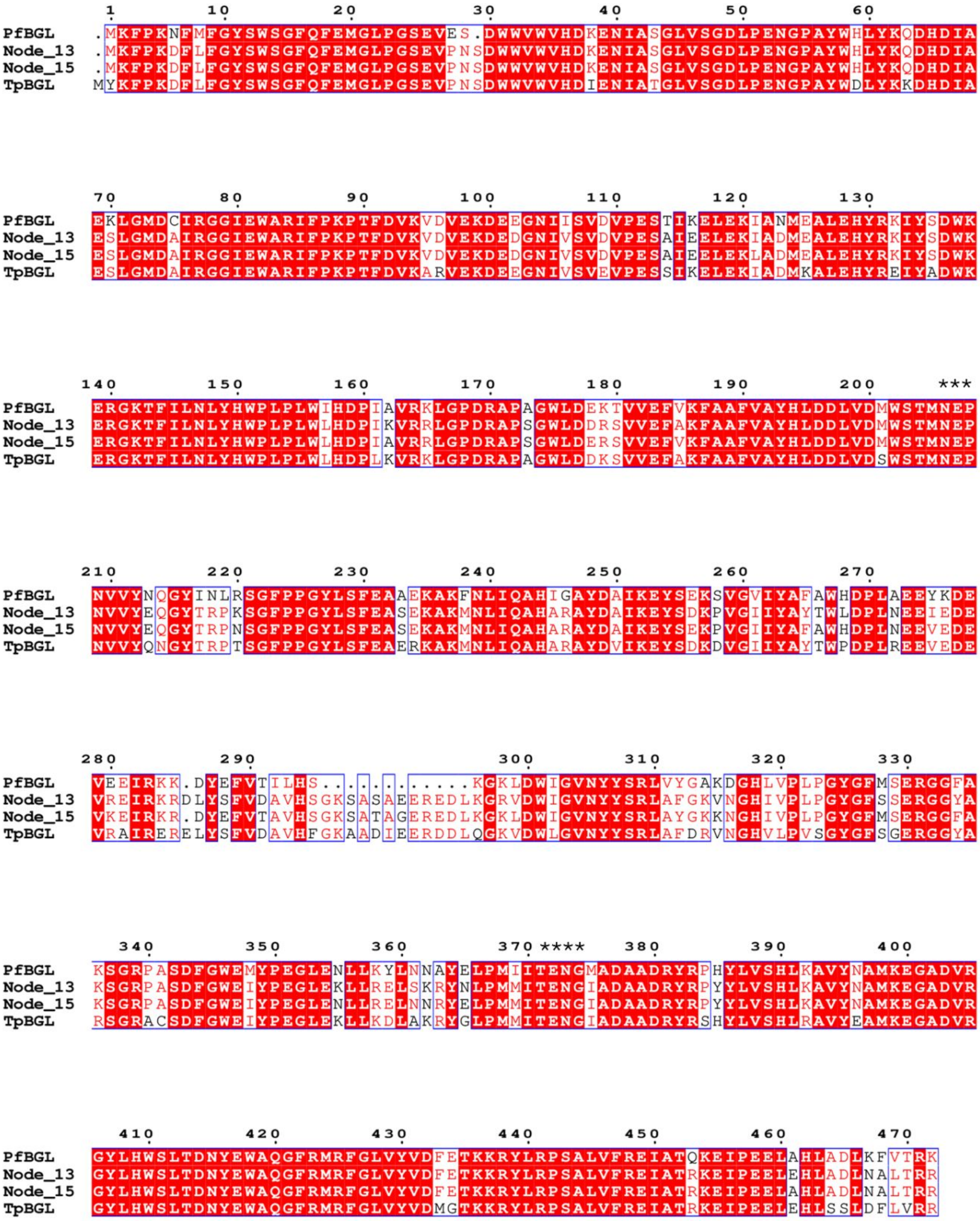

Figure S12. Amino acid sequence alignments of Nodes 13 and 15, PfBGL and TpBGL. The NEP and TENG sequence motifs are indicated by asterisks. The multiple sequence alignments were performed using ClustalW[16] and visualized with ESPript 3.[17]

REFERENCES

(1) BRENDA Enzyme Database, https://www.brenda-enzymes.org/, Accessed in March 2025.
(2) Erkanli, M. E.; El-Halabi, K.; Kang, T. K.; Kim, J. R. Hotspot Wizard-informed engineering of a hyperthermophilic beta-glucosidase for enhanced enzyme activity at low temperatures. *Biotechnol Bioeng* **2024**, *121* (7), 2079-2090.
(3) Kroll, A.; Rousset, Y.; Hu, X. P.; Liebrand, N. A.; Lercher, M. J. Turnover number predictions for kinetically uncharacterized enzymes using machine and deep learning. *Nat Commun* **2023**, *14* (1), 4139.
(4) Yu, H.; Deng, H.; He, J.; Keasling, J. D.; Luo, X. UniKP: a unified framework for the prediction of enzyme kinetic parameters. *Nat Commun* **2023**, *14* (1), 8211.
(5) Kroll, A.; Lercher, M. J. DLKcat cannot predict meaningful k (cat) values for mutants and unfamiliar enzymes. *Biol Methods Protoc* **2024**, *9* (1), bpae061.
(6) Arcus, V. L.; Prentice, E. J.; Hobbs, J. K.; Mulholland, A. J.; Van der Kamp, M. W.; Pudney, C. R.; Parker, E. J.; Schipper, L. A. On the Temperature Dependence of Enzyme-Catalyzed Rates. *Biochemistry* **2016**, *55* (12), 1681-1688.
(7) Sakaguchi, M.; Osaku, K.; Maejima, S.; Ohno, N.; Sugahara, Y.; Oyama, F.; Kawakita, M. Highly conserved salt bridge stabilizes a proteinase K subfamily enzyme, Aqualysin I, from Thermus aquaticus YT-1. *AMB Express* **2014**, *4*, 59.
(8) Akiba, T.; Sano, S.; Yanase, T.; Ohta, T.; Koyama, M. Optuna: A Next-generation Hyperparameter Optimization Framework. *Kdd'19: Proceedings of the 25th Acm Sigkdd International Conferencce on Knowledge Discovery and Data Mining* **2019**, 2623-2631.
(9) Tamura, K.; Stecher, G.; Kumar, S. MEGA11: Molecular Evolutionary Genetics Analysis Version 11. *Mol Biol Evol* **2021**, *38* (7), 3022-3027.
(10) Le, S. Q.; Gascuel, O. An improved general amino acid replacement matrix. *Mol Biol Evol* **2008**, *25* (7), 1307-1320.
(11) Barruetabeña, N.; Alonso-Lerma, B.; Galera-Prat, A.; Joudeh, N.; Barandiaran, L.; Aldazabal, L.; Arbulu, M.; Alcalde, M.; De Sancho, D.; Gavira, J. A.; et al. Resurrection of efficient Precambrian endoglucanases for lignocellulosic biomass hydrolysis. *Commun Chem* **2019**, *2*, 76.
(12) Hon, J.; Marusiak, M.; Martinek, T.; Kunka, A.; Zendulka, J.; Bednar, D.; Damborsky, J. SoluProt: prediction of soluble protein expression in Escherichia coli. *Bioinformatics* **2021**, *37* (1), 23-28.
(13) Jung, F.; Frey, K.; Zimmer, D.; Muhlhaus, T. DeepSTABp: A Deep Learning Approach for the Prediction of Thermal Protein Stability. *Int J Mol Sci* **2023**, *24* (8), 7444.
(14) Maki, M. L.; Armstrong, L.; Leung, K. T.; Qin, W. Increased expression of beta-glucosidase A in Clostridium thermocellum 27405 significantly increases cellulase activity. *Bioengineered* **2013**, *4* (1), 15-20.
(15) Jordan, D. B.; Braker, J. D. Rate-limiting steps of a stereochemistry retaining beta-d-xylosidase from Geobacillus stearothermophilus acting on four substrates. *Arch Biochem Biophys* **2015**, *583*, 73-78.
(16) Larkin, M. A.; Blackshields, G.; Brown, N. P.; Chenna, R.; McGettigan, P. A.; McWilliam, H.; Valentin, F.; Wallace, I. M.; Wilm, A.; Lopez, R.; et al. Clustal W and Clustal X version 2.0. *Bioinformatics* **2007**, *23* (21), 2947-2948.
(17) Robert, X.; Gouet, P. Deciphering key features in protein structures with the new ENDscript server. *Nucleic Acids Res* **2014**, *42* (Web Server issue), W320-324.
